# The "double-edged sword" effect of non-steroidal anti-inflammatory drugs (NSAIDs) in the treatment of endometriosis (EMS)

**DOI:** 10.1186/s12958-025-01508-7

**Published:** 2025-12-06

**Authors:** Zunlin Shi, Zhi Li, Yirou Li, Fan Yang

**Affiliations:** 1 College of Electronics and Information Engineering, University of Sichuan, Chengdu, Sichuan Province 610065 China; 2 Department of Gynecology and Obstetrics, West China Second Hospital, University of Sichuan, Chengdu, Sichuan Province 610041 China; 3 Department of Gynecology and Obstetrics, Development and Related Diseases of Women and Children Key Laboratory of Sichuan Province, Key Laboratory of Birth Defects and Related Diseases of Women and Children, Ministry of Education, West China Second Hospital, Sichuan University, Chengdu, Sichuan 610041 P.R. China

**Keywords:** Endometriosis, NSAIDs, Mendelian randomization, Network toxicology, Molecular docking, Drug safety

## Abstract

**Background:**

Non-steroidal anti-inflammatory drugs (NSAIDs) are commonly used to alleviate pain associated with endometriosis (EMS), yet the impact of their long-term use on disease progression remains unclear. This study investigates the dual role of NSAIDs in EMS pathogenesis using network toxicology and Mendelian randomization (MR).

**Methods:**

The toxicity and ADMET profiles of nine NSAIDs were screened using ProTox 3.0 and ADMETlab 2.0. Potential drug targets were predicted using PharmMapper, STITCH, and SwissTargetPrediction, while EMS-related targets were retrieved from GeneCards, OMIM, and CTD databases. For the MR analysis, cis-eQTLs for whole blood tissue from GTEx v8 served as instrumental variables, based on the inflammatory nature of endometriosis. Outcome data were from an independent GWAS summary dataset (19,588 cases, 213,669 controls). Our analysis adhered to MR independence assumptions, ensuring no sample overlap between exposure and outcome data. Functional enrichment and molecular docking explored the underlying mechanisms.

**Results:**

By integrating drug targets with disease genes, we first identified 463 overlapping targets. We revealed that *EPHB4* is a core hub mediating the potential “risk-promoting” effects of nearly all NSAIDs, with its functions enriched in key pathological processes such as angiogenesis. Molecular docking confirmed that eight NSAIDs could stably bind to *EPHB4*. Crucially, we found that indomethacin exhibited a unique “dual-regulatory” pattern: it simultaneously targeted the protective target *PTGER4* and the risk-associated target *EPHB4*. Molecular docking further substantiated, at the atomic level, that indomethacin possesses strong binding affinity for both targets, providing a structural biology explanation for its observed genetic effects.

**Interpretation:**

This study provides, for the first time, robust causal evidence from both genetic and structural biology perspectives for the “double-edged sword” attribute of NSAIDs in EMS, and proposes a new paradigm of “target-oriented heterogeneous effects.” We found that specific NSAIDs might inadvertently promote disease progression by activating the *EPHB4* pathway, while indomethacin stands out as a key exception due to its unique dual-action mechanism. These findings not only offer a critical explanation for the current clinical controversy but also lay a solid scientific foundation for advancing EMS management from “empirical medication” towards “precision-based selection guided by molecular mechanisms.”

**Graphical abstract:**

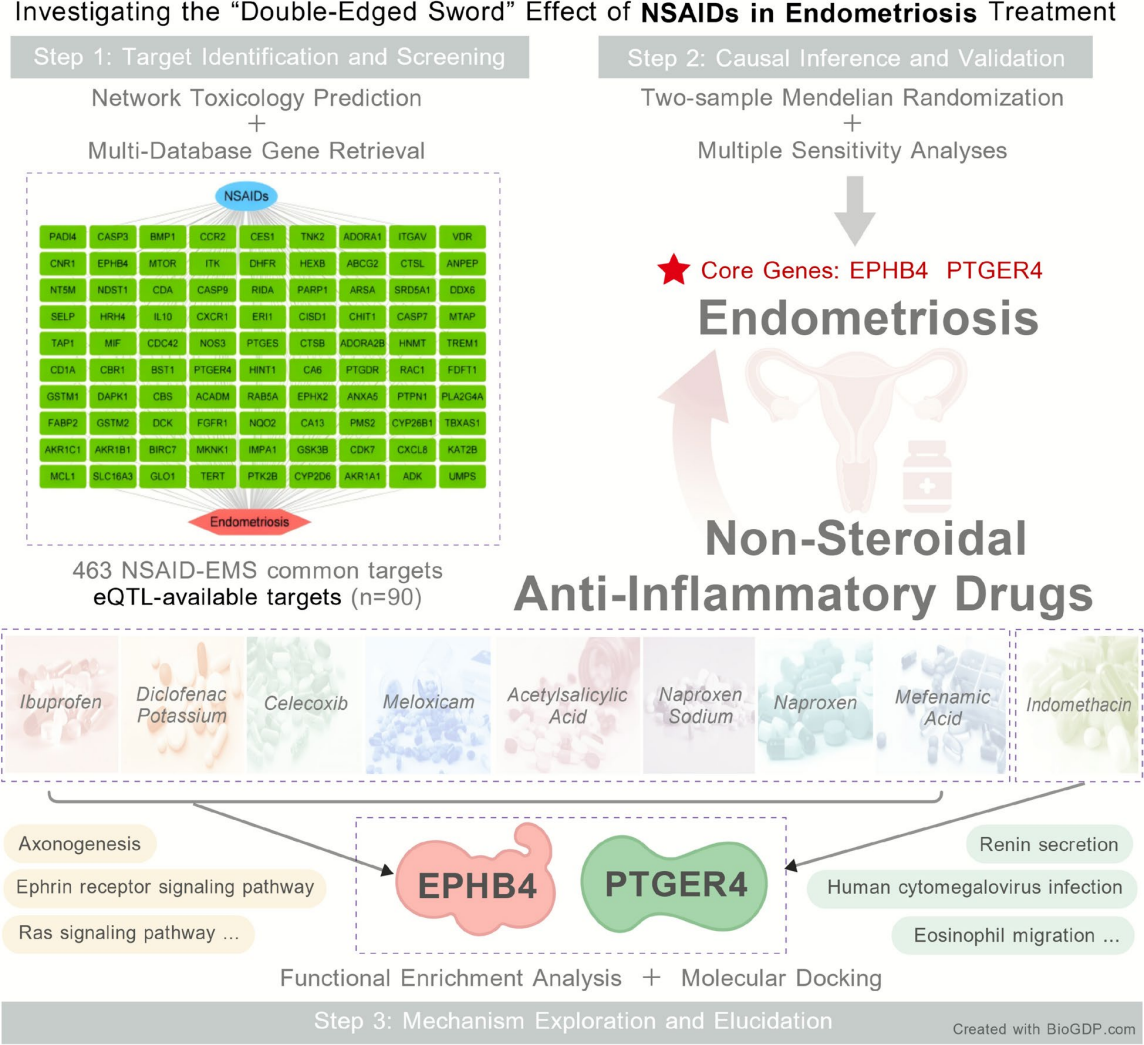

**Supplementary Information:**

The online version contains supplementary material available at 10.1186/s12958-025-01508-7.

## Highlights

1. Proposing a New Paradigm: By constructing a multi-dimensional analytical framework that integrates network toxicology, causal inference, and structural biology, this study is the first to reveal the “target-oriented heterogeneous effects” of NSAIDs in the treatment of dysmenorrhea caused by endometriosis, challenging the traditional “one-size-fits-all” treatment model.
2. Identifying Key Targets: This study established a complete evidence chain from “target screening” to “causal inference” and finally to “structural validation,” identifying for the first time *EPHB4* as the central hub gene mediating the potential risk effects of NSAIDs and elucidating that its function is primarily achieved through angiogenesis pathways.
3. Elucidating a Complex Mechanism: Through cross-validation between genetic causality and molecular structural simulation, we elucidated the unique “dual regulation” mode of indomethacin: mediating a protective effect via *PTGER4* while simultaneously mediating a potential risk via *EPHB4*.
4. Providing Structural Evidence: Molecular docking simulations provided structural biology evidence at the atomic level for the aforementioned genetic effects, confirming that indomethacin possesses high-affinity binding potential with both *PTGER4* and *EPHB4*.
5. Guiding Precision Medication: This study provides a computationally driven, critical basis for the precision medication of endometriosis. It not only offers theoretical support for considering indomethacin as a preferred option but also points the way for the development of novel NSAIDs that avoid the *EPHB4* pathway.

**Supplementary Information:**

The online version contains supplementary material available at 10.1186/s12958-025-01508-7.

## Introduction

Endometriosis (EMS) is a chronic inflammatory disease affecting approximately 10% of reproductive-aged women globally, characterized by the abnormal growth of endometrial-like tissue outside the uterine cavity [1, 2]. Beyond causing severe pelvic pain and infertility, significant burdens to patients, the disease also imposes substantial socioeconomic costs [3, 4]. EMS not only significantly impacts female reproductive health, but its complex pathophysiology also involves sustained inflammatory responses, angiogenesis, and tissue invasion, processes that collectively drive disease progression and recurrence [5–7].

In clinical practice, pain management is central to EMS treatment. Non-steroidal anti-inflammatory drugs (NSAIDs), such as ibuprofen and paracetamol, are first-line medications for alleviating EMS-related symptoms, particularly dysmenorrhea, due to their established anti-inflammatory and analgesic effects [8–10]. These drugs are widely used globally, especially for alleviating gynecological symptoms. Their primary mechanism of action involves inhibiting cyclooxygenase (COX) enzymes, thereby reducing prostaglandin synthesis [11, 12]. However, despite the widely recognized analgesic efficacy of NSAIDs, the potential impact of their long-term use on disease progression itself remains an unresolved question [11]. Preliminary studies suggest that NSAIDs may exert complex effects on EMS that extend beyond mere pain relief by influencing key pathological processes such as inflammation and angiogenesis [13–15]. Nevertheless, these studies often lack systematic assessment, leaving the understanding of the “double-edged sword” effect of NSAIDs in EMS—that is, the potential for concurrent benefits and risks—at a relatively ambiguous level.

Given the substantial disease burden of endometriosis and the widespread use of non-steroidal anti-inflammatory drugs (NSAIDs), their long-term safety, particularly the potential impact on disease progression, represents a critical and unresolved clinical challenge. However, existing research lacks a systematic assessment of the complex mechanisms potentially underlying the effects of NSAIDs in EMS, and the understanding of their “double-edged sword” effect has long remained at a relatively ambiguous level. To address this, our study strategically integrates network toxicology with Mendelian randomization (MR) analysis for the first time, aiming to construct a multi-layered, experimentally verifiable scientific hypothesis framework for this mechanistically ambiguous “double-edged sword” effect of NSAIDs in EMS. The advantage of this integrated strategy lies in its ability to overcome the limitations of single-method approaches: network toxicology systematically reveals potential molecular networks, while MR analysis provides causal inference from a genetic perspective, effectively circumventing the confounding biases inherent in traditional observational studies. The combination of these methods is poised to transform the macroscopic clinical phenomenon of the “double-edged sword” into a series of testable scientific hypotheses concerning specific molecular targets and pathways. Specifically, this study will first identify potential targets of NSAIDs, then use MR analysis to infer the causal relationship between these targets and EMS risk at the population level, and finally provide structural biology evidence at the atomic level through molecular docking simulations. We anticipate that this research will not only construct a potential molecular mechanism model of how NSAIDs influence EMS risk, but more importantly, it will provide a series of directly testable molecular targets to resolve this clinical controversy, thereby laying a crucial scientific foundation for optimizing clinical management strategies and guiding subsequent wet-lab validation.

## Materials and methods

**Fig. 1:**
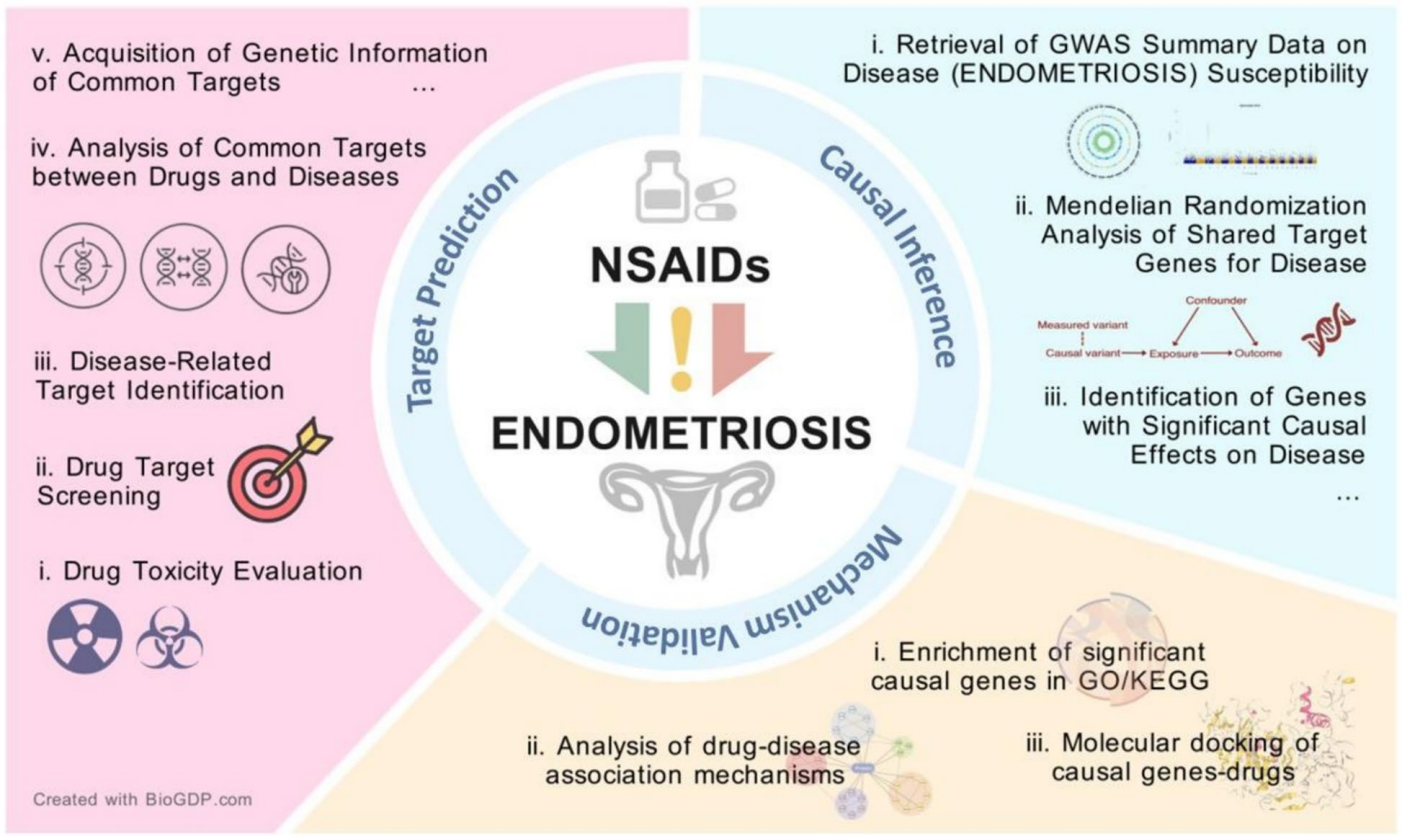
Schematic diagram of the combined network toxicology and MR analysis workflow for assessing NSAIDs’ impact on female reproductive metabolic health

This research utilizes a novel interdisciplinary approach that integrates network toxicology with Mendelian randomization (MR) analysis, aiming to investigate the influence of non-steroidal anti-inflammatory drugs (NSAIDs) on the reproductive metabolic health of women, particularly concerning their potential association with endometriosis (refer to Fig. 1).

### Network toxicology assessment of NSAIDs and endometriosis-related potential targets

In order to comprehensively assess the global influence of NSAIDs on women's reproductive metabolic well-being, particularly their possible role in exacerbating endometriosis, this study employed network toxicology methodologies to systematically identify potential NSAID targets associated with endometriosis [16].

Initially, we meticulously compiled and synthesized data concerning various NSAIDs utilized worldwide for managing dysmenorrhea, concentrating on nine representative medications listed in Table 1: Acetylsalicylic acid, Celecoxib, Diclofenac potassium, Ibuprofen, Indomethacin, Mefenamic acid, Meloxicam, NAPROXEN, and Naproxen sodium. Throughout this process, we adhered to several international guidelines and databases to ensure the accuracy and comprehensiveness of the information gathered [17–24].

**Table 1:** Information on NSAIDs used for dysmenorrhea treatment worldwide

Drug Name	Class	Dosage Form	Typical Dosage (for Dysmenorrhea)	Common Side Effects	Contraindications	Price (USD, Approximate, Varies by Region)	Commonly Used Regions
Acetylsalicylic Acid (Aspirin) (CAS: 50–78-2)	Salicylic acid derivatives	Tablet, Chewable	300–600 mg every 4–6 h	Nausea, Vomiting, Heartburn, Tinnitus, Increased bleeding risk	Hypersensitivity, Peptic ulcer disease, Severe liver or kidney disease, Children/Teenagers with viral infections (Reye’s syndrome risk), Hemophilia, Gout, Late pregnancy	$0.01 -$0.10 per tablet	Global, but usage varies by region depending on availability and guidelines. More common in regions with established healthcare systems
Celecoxib (Celebrex) (CAS: 169,590–42-5)	Cyclooxygenase-2 (COX-2) inhibitors	Capsule	100–200 mg every 12 h	Diarrhea, Gas, Nausea, Headache, Dizziness	Hypersensitivity, Peptic ulcer disease, Severe liver or kidney disease, Pregnancy (third trimester), Cardiac disease, Hypertension	$1 -$3 per capsule	North America, Europe, parts of Asia
Diclofenac Potassium (Voltaren Rapide) (CAS: 15,307–81-0)	Acetic acid derivatives (including indole derivatives)	Tablet, Liquid Gel	50 mg every 8 h	Nausea, Vomiting, Diarrhea, Headache, Dizziness, Abdominal pain	Hypersensitivity, Peptic ulcer disease, Severe liver or kidney disease, Pregnancy (third trimester), Inflammatory bowel disease, Heart failure	$0.10 -$0.50 per tablet	Global, widely used in Europe, South America, and parts of Asia
Ibuprofen (Advil, Motrin) (CAS: 15,687–27-1)	Propionic acid derivatives	Tablet, Capsule, Liquid	200–400 mg every 4–6 h	Nausea, Vomiting, Headache, Dizziness, Heartburn, Rash	Hypersensitivity, Peptic ulcer disease, Severe liver or kidney disease, Pregnancy (third trimester), Asthma (in some individuals)	$0.05 -$0.30 per tablet	Global, one of the most commonly used NSAIDs worldwide
Indomethacin (Indocid) (CAS: 53–86-1)	Acetic acid derivatives (including indole derivatives)	Capsule, Extended-release Capsule	25–50 mg 2–3 times daily	Nausea, Vomiting, Headache, Dizziness, Indigestion, Heartburn	Hypersensitivity, Peptic ulcer disease, Severe liver or kidney disease, Pregnancy (third trimester), Asthma, Parkinson’s disease (worsening of symptoms), Children < 14 years old (not recommended)	$0.10 -$0.60 per capsule	Less common globally compared to others, used in specific indications
Mefenamic Acid (Ponstan, Mefenamic) (CAS: 61–68-7)	Fenamates	Capsule, Tablet	250–500 mg every 6 h	Nausea, Vomiting, Diarrhea, Headache, Dizziness, Rash	Hypersensitivity, Peptic ulcer disease, Severe liver or kidney disease, Pregnancy (third trimester), Asthma (in some individuals)	$0.20 -$0.80 per capsule	More common in Europe, South America, and parts of Asia
Meloxicam (Mobicox) (CAS: 71,125–38-7)	Oxicams	Tablet, Oral Suspension	7.5–15.5 mg once daily	Diarrhea, Gas, Nausea, Dizziness, Headache, Constipation	Hypersensitivity, Peptic ulcer disease, Severe liver or kidney disease, Pregnancy (third trimester), Inflammatory bowel disease, Heart failure	$0.50 -$2 per tablet	Global, widely used in Europe, North America, and parts of Asia
Naproxen (Naprosyn) (CAS: 303–53-4)	Propionic acid derivatives	Tablet, Capsule	250–500 mg every 6–8 h	Nausea, Vomiting, Headache, Dizziness, Heartburn, Constipation	Hypersensitivity, Peptic ulcer disease, Severe liver or kidney disease, Pregnancy (third trimester), Asthma (in some individuals)	$0.10 -$0.50 per tablet	Global, widely used in North America, Europe, and parts of Asia
Naproxen Sodium (Anaprox) (CAS: 26,159–34-2)	Propionic acid derivatives	Tablet, Extended-release Tablet	220 mg every 8–12 h (immediate-release) 440–880 mg once daily (extended-release)	Nausea, Vomiting, Headache, Dizziness, Heartburn, Constipation	Hypersensitivity, Peptic ulcer disease, Severe liver or kidney disease, Pregnancy (third trimester), Asthma (in some individuals)	$0.20 -$0.70 per tablet	Global, widely used in North America, Europe, and parts of Asia

The toxicity and ADMET profiles of the drugs were evaluated using the ProTox 3.0 and ADMETlab 2.0 platforms [25, 26]. Potential targets for these drugs were predicted through pharmacophore matching (PharmMapper), biological interaction networks (STITCH), and machine learning algorithms (SwissTargetPrediction) [27–33]. Concurrently, EMS-related genes were retrieved from the GeneCards website, the OMIM database, and the Comparative Toxicogenomics Database (CTD) using “endometriosis” as the keyword [34–40]. By comparing the NSAID targets with the EMS-related genes, we identified the overlapping target genes, which served as the subjects for the subsequent Mendelian randomization analysis (see Table 2 for detailed information on the databases and platforms).

**Table 2:** Data resource table

Data Type	Database/Platform	URL	Search Terms	Purpose
NSAIDs drug information	Public data worldwide	/	Dysmenorrhea, NSAIDs	Screen NSAIDs drugs related to dysmenorrhea treatment
Toxicity profiles	ProTox 3.0	https://tox.charite.de/protox3/	Acetylsalicylic acid, Celecoxib, Diclofenac potassium, Ibuprofen, Indomethacin, Mefenamic acid, Meloxicam, NAPROXEN, Naproxen sodium	Evaluate the toxicity profiles of NSAIDs
ADME properties	ADMETlab 2.0	https://admet.scbdd.com/		Evaluate the ADME properties and toxicity of NSAIDs
Target prediction	PharmMapper	http://lilab.ecust.edu.cn/pharmmapper	Acetylsalicylic acid, Celecoxib, Diclofenac potassium, Ibuprofen, Indomethacin, Mefenamic acid, Meloxicam, NAPROXEN, Naproxen sodium	Predict potential targets of NSAIDs based on pharmacophore matching
Target prediction	STITCH	http://stitch.embl.de/		Predict potential targets of NSAIDs based on biological interaction networks
Target prediction	SwissTargetPrediction	http://www.swisstargetprediction.ch/		Predict potential targets of NSAIDs based on machine learning algorithms
Disease targets	CTD	https://ctdbase.org/	endometriosis	Identify targets associated with endometriosis
Disease targets	GeneCards	https://www.genecards.org/		Identify targets associated with endometriosis
Disease targets	OMIM	https://omim.org/		Identify targets associated with endometriosis

### Two-sample Mendelian randomization analysis

To investigate the potential causal relationship between the expression of NSAID target genes and the risk of endometriosis, we employed a two-sample Mendelian randomization (MR) framework. This design utilizes genetic variants as instrumental variables (IVs) to effectively overcome confounding bias and reverse causation inherent in traditional observational studies.

#### Data sources

Instrumental variables for the exposure data were sourced from the GTEx Portal v8 database (Supplementary Data 1) [41–44]. Considering the chronic inflammatory pathology of endometriosis and the primary mechanism of NSAIDs, which involves modulating inflammatory mediators in the bloodstream, we selected cis-eQTL (expression quantitative trait loci) data from whole blood tissue. This data was derived from the European (EUR) ancestry subset of the GTEx v8 cohort, comprising 715 donors. We screened for SNPs significantly associated with target gene expression (*p* < 1 × 10⁻^5^) as potential IVs. For the outcome data, the genome-wide association study (GWAS) summary statistics for endometriosis were obtained from the NHGRI-EBI database (ID: GCST90454213). This dataset integrates data from the Estonian Biobank and the FinnGen study, encompassing 19,588 cases and 213,669 controls of European ancestry (Supplementary Data 2) [45].

#### Instrumental variable selection, data harmonization, and statistical power assessment

For each target gene, we extracted eligible SNPs from the GTEx eQTL data to serve as instrumental variables (IVs). We performed linkage disequilibrium (LD) clumping using PLINK software (v1.9) with the following parameters: an LD window size of 100 kb and an r^2^ threshold of 0.1 within the window. We set the clumping p-value threshold (clump_p) to 1 to ensure that all candidate SNPs passing the initial screening were included in the clumping process, thereby maximizing the retention of independent IVs [46, 47]. Subsequently, we harmonized the exposure and outcome datasets using the TwoSampleMR package in R to align effect and reference alleles, removing any incompatible SNPs. We confirmed that the exposure data (GTEx) and outcome data (GCST90454213) were derived from completely independent study cohorts with no sample overlap, thus avoiding sample overlap bias [48, 49]. All subsequent analyses, including IV strength assessment and power calculations, were based on the harmonized final set of IVs. The strength of the genetic IVs was assessed using the F-statistic, calculated as F = R^2^(n—2)/(1—R^2^), where n represents the effective GWAS sample size for the SNP, and R^2^ represents the SNP’s predictive power for target gene expression. We calculated the F-statistics and ensured that all SNPs included in the analysis had an F-statistic greater than 10 to exclude weak instrument bias [50].

Furthermore, to quantify the statistical sensitivity of our study design, we conducted a precise statistical power assessment for each target gene that passed the aforementioned screening and harmonization [51]. This assessment aimed to determine the minimum causal effect that our study design could detect with 80% power. The specific calculation procedure was as follows: First, for each target gene, we calculated its average explained variance (R^2^) and mean F-statistic based on the harmonized final IV set. We employed the non-central chi-squared distribution method, as described by Brion et al. [51], for power calculation. The core of this method involves calculating a non-centrality parameter (λ), with the formula as follows: (1).

1 $$\boldsymbol N\boldsymbol C\boldsymbol P=\frac{\boldsymbol N\times\boldsymbol R^2\times\left(\ln\left(\boldsymbol O\boldsymbol R\right)\right)^2}{1-\boldsymbol R^2}$$

where N is the total outcome sample size (233,257), R^2^ is the average explained variance of the IVs, and OR is the assumed causal effect size. This function takes N, R^2^, the significance level (α = 0.05), and an assumed OR value as input to output the corresponding statistical power. Finally, to precisely identify the minimum OR value corresponding to 80% power, we implemented a bisection search algorithm. This algorithm iteratively narrows down a predefined range of OR values to approach the target power until a preset precision of 0.001 is achieved. The resulting OR value represents the minimum detectable effect (MDE) for that target gene under our study design. This series of assessments provides a crucial quantitative benchmark for interpreting the causal effect estimates for each gene in the Results section. We recognize that the initial screening may introduce potential biases; therefore, we have implemented a series of rigorous sensitivity analyses and validation steps to ensure the robustness of our final findings.

#### Primary MR analysis and multi-method consistency testing

Our primary causal estimation was performed using the Inverse-Variance Weighted (IVW) method, which is the most efficient and precise estimator under the assumption that all instrumental variables are valid (i.e., the absence of horizontal pleiotropy) [52]. To verify the robustness of our results, we employed a series of complementary MR methods for cross-validation, including the Weighted Median method (which can provide consistent estimates even if up to 50% of the IVs are invalid) and MR-Egger regression [53, 54].

#### Systematic detection of horizontal pleiotropy

Horizontal pleiotropy, where an IV influences the outcome through pathways other than the exposure, is a core challenge to the assumptions of MR analysis. We conducted a systematic assessment for this. First, we employed MR-Egger regression, where the intercept term provides a direct test for unbalanced horizontal pleiotropy; an intercept significantly deviating from zero indicates directional pleiotropic bias [55, 56]. Second, we applied the MR-PRESSO (Pleiotropy RESidual Sum and Outlier) method to identify and correct for outlier SNPs potentially driven by horizontal pleiotropy, providing MR results after removal of these outliers to assess their impact on the overall causal estimate [57, 58]. Finally, and most critically, we performed colocalization analysis. This is a decisive step for ruling out spurious associations caused by linkage disequilibrium (LD), a major form of horizontal pleiotropy. Therefore, for all genes showing a significant causal association in the MR analysis, we further conducted colocalization analysis to directly test whether the genetic signal influencing gene expression and the genetic signal influencing disease risk share the same causal variant [59]. Only when evidence supported colocalization could we largely rule out spurious associations, thereby strengthening the credibility of our causal inference.

#### Heterogeneity and robustness testing

For heterogeneity testing, we used Cochran’s Q statistic to assess the heterogeneity in the effect estimates of the IVs. Significant heterogeneity may suggest the presence of horizontal pleiotropy or other model misspecifications [50, 60]. Additionally, we performed a leave-one-out analysis to determine if the overall causal association was driven by a single SNP with a strong effect, thereby evaluating the robustness of our results [50, 61].

#### Causal direction and model validation

To further confirm the direction of causality, we adopted two complementary validation methods. First, we performed Steiger directionality testing to confirm that the variance in the exposure (gene expression) explained by the genetic variants (R^2^) was greater than the variance they explained in the outcome (disease risk), thus statistically ruling out the possibility of reverse causation [50, 62, 63]. Second, we conducted a reverse Mendelian randomization analysis, using SNPs significantly associated with the outcome (endometriosis) as IVs to test their effect on the exposure (target gene expression) [50]. In this analysis, for all genes that were significant in the forward direction, the reverse MR analysis could not be performed due to an insufficient number of IVs. This result itself provides important information, as the lack of genetic evidence for a reverse causal relationship lends support to our primary findings.

#### Multiple testing correction and Bayesian model validation

As we tested multiple target genes, we applied Bonferroni correction and the Benjamini-Hochberg (BH) procedure for False Discovery Rate (FDR) correction to control the overall false positive rate [64, 65]. Furthermore, to validate our results within a Bayesian framework, we employed Bayesian Weighted Mendelian Randomization (BWMR) analysis [66]. Since there were no overlapping SNPs among the eQTL instruments for different NSAID target genes, precluding a multivariate MR (MVMR) analysis, BWMR served as a rigorous univariate Bayesian method, providing us with an additional layer of validation based on a different statistical framework.

Through this comprehensive workflow, spanning from initial screening to multi-faceted validation, we ensured that the final causal conclusions are robust and credible, even though relatively lenient thresholds were used in the initial screening phase.

### Functional enrichment analysis

To interpret the findings from the Mendelian randomization analysis from a biological functional perspective, we designed a functional enrichment analysis workflow. This analysis prioritized candidate genes with the most reliable causal evidence, as validated by multiple sensitivity analyses and colocalization analysis. Acknowledging the limitations of pathway enrichment analysis based on a single gene, we adopted a network expansion strategy. Specifically, we used the core causal gene, *EPHB4*, as a seed to construct a gene network comprising its direct interacting proteins using the STRING database [67]. Subsequently, we utilized the clusterProfiler package in R to perform KEGG pathway and Gene Ontology (GO) analyses on all genes within this network, with the latter encompassing the three dimensions of Biological Process (BP), Molecular Function (MF), and Cellular Component (CC) [68, 69]. The significance of enrichment was assessed using a hypergeometric test, and *p*-values were corrected for multiple testing using the Benjamini-Hochberg (BH) method. Terms with a False Discovery Rate (FDR) less than 0.05 were defined as significantly enriched [64]. To intuitively visualize the results, we employed visualization tools such as bubble plots and bar charts. Through this analysis, we aimed to elucidate the biological context and key pathways involving the core causal target, providing a molecular-level explanation for the mechanism of action of non-steroidal anti-inflammatory drugs (NSAIDs) in endometriosis.

### Molecular docking and mechanistic validation

To validate and elucidate the interactions between non-steroidal anti-inflammatory drugs (NSAIDs) and our identified key targets at the atomic level, we performed molecular docking analysis. This analysis aimed to predict the binding modes and affinities of the drugs to the target proteins, providing structural biology evidence for the observed causal associations. The targets for molecular docking were limited to proteins encoded by genes that passed all our MR sensitivity tests (including heterogeneity and pleiotropy tests) and were validated by colocalization analysis. The three-dimensional (3D) structures of these targets were retrieved from the Protein Data Bank (PDB) [70]. Prior to docking, all protein structures underwent standard preprocessing, which included removing water molecules, native ligands, and metal ions, followed by the addition of hydrogen atoms to ensure structural integrity and computational accuracy.

Molecular docking calculations were performed using the online server CB-Dock2 [71]. This platform automatically identifies potential binding pockets on the protein surface and performs docking while considering the flexibility of both the protein and the ligand [72]. We used its built-in scoring function, based on AutoDock Vina, to assess binding affinity. This score, reported in kcal/mol, is more negative for stronger, more stable binding [73]. The analysis of docking results focused on the following metrics: first, binding affinity, where we compared the binding strengths of different NSAIDs to the same target and the same NSAID to different targets; second, binding conformation and interactions, where we visually analyzed the ligand’s pose within the binding pocket and identified key interaction forces, such as hydrogen bonds, hydrophobic interactions, and salt bridges. Through these analyses, we aim to provide an intuitive structural biology explanation for the central question of why different NSAIDs exert varying effects on endometriosis risk, thereby advancing the causal associations discovered through genetics to the specific mechanistic level of molecular interactions.

### Ethics statement

This study utilized publicly available and de-identified data from GWAS and bioinformatics databases. No new human or animal experiments were conducted. Therefore, formal ethics approval was not required. All data sources are cited and comply with the original data access agreements.

### Statistical analysis

All statistical analyses were performed using R software (version 4.2.3). The Mendelian randomization analyses were primarily conducted using the TwoSampleMR package (version 0.6.8), and colocalization analysis was performed using the coloc package. Functional enrichment analysis was carried out with the clusterProfiler package. Network construction and molecular docking were performed using the STRING database and the CB-Dock2 online server, respectively. Statistical significance was defined as a two-sided *p*-value < 0.05. For analyses involving multiple testing, such as MR and enrichment analysis, the False Discovery Rate (FDR) was controlled, and an FDR < 0.05 was considered statistically significant.

### Role of the funder

This study was financially supported by the National Key Research and Development Program of China (Grant No. 2022YFC2704103). The funder had no role in study design, data collection, analysis, interpretation, or writing of the report.

## Results

### Overview of analyzed NSAIDs and their target genes

**Fig. 2:**
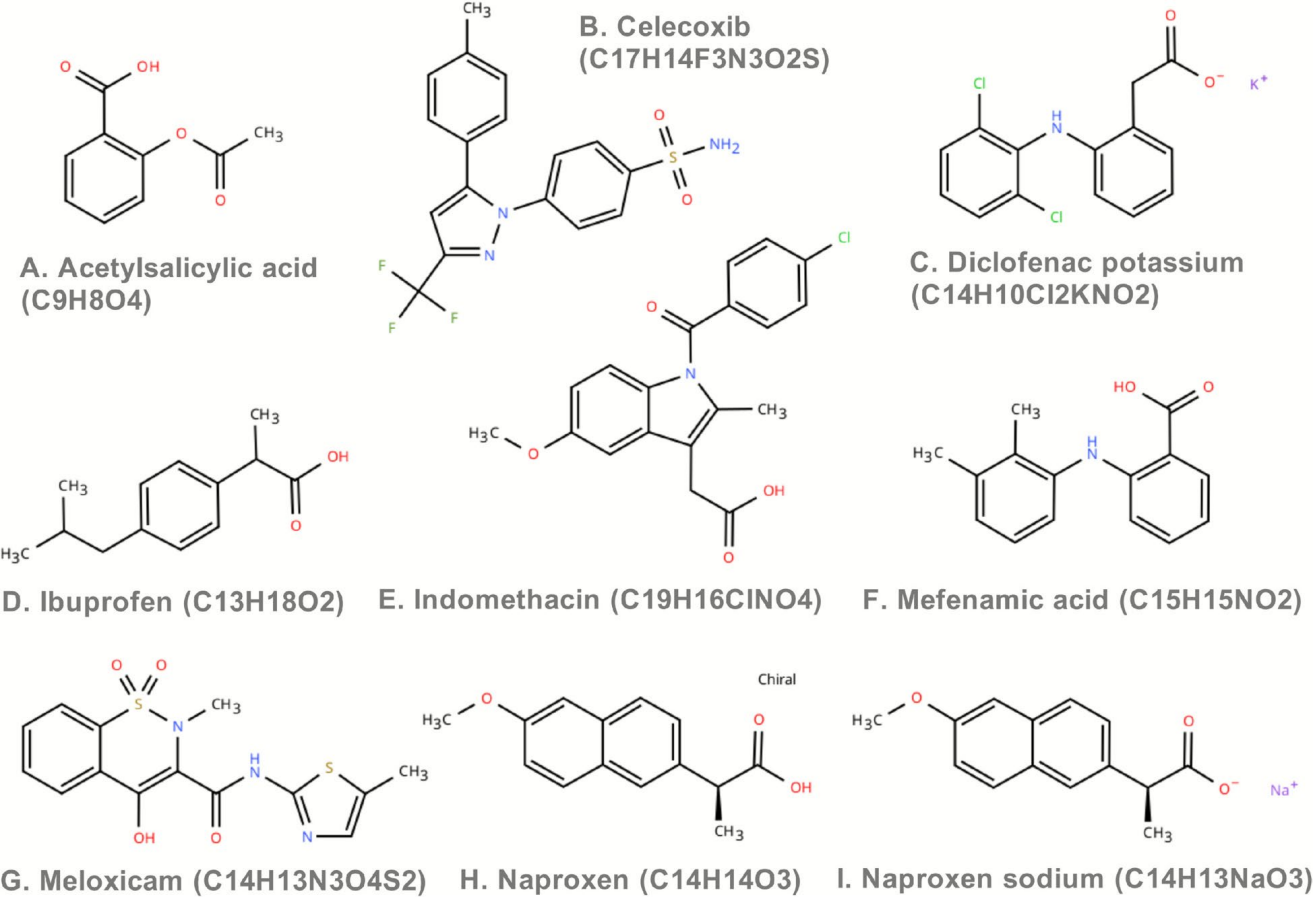
Nine structures of non-steroidal anti-inflammatory drugs (NSAIDs)

This study focuses on nine widely used non-steroidal anti-inflammatory drugs (NSAIDs): acetylsalicylic acid, celecoxib, diclofenac potassium, ibuprofen, indomethacin, mefenamic acid, meloxicam, naproxen, and naproxen sodium (Fig. 2). Clinically, these drugs are primarily used for analgesia, antipyresis, and anti-inflammatory effects, acting classically through the inhibition of cyclooxygenases (COX), particularly COX-1 and COX-2; thereby reducing the synthesis of inflammatory mediators, like prostaglandins. However, they may also exert effects through other pathways, such as to influence alternative routes of arachidonic acid metabolism, to disrupt cellular signaling pathways, or to alter gene expression (Table 3). Due to their efficacy, these NSAIDs have been globally recommended, particularly as first-line treatments for dysmenorrhea associated with endometriosis. However, despite their widespread use, their long-term use in specific populations may be associated with potential adverse effects, particularly on the reproductive and metabolic systems, which prompts further investigation into their potential risks.

**Table 3:** Toxicity prediction for nine NSAIDs

NSAIDs	ADMETlab 2.0 Database Prediction						ProTox 3.0 Database Prediction
	PPB	CL	Rat Oral Acute Toxicity	NR-PPAR-gamma	CYP2C9 inhibitor	CYP2C9 substrate	Cytochrome CYP2C9
Acetylsalicylic acid	59.41%	2.736	0.756	0.007	0.059	0.143	Inactive
Celecoxib	94.96%	0.992	0.771	0.008	0.858	0.696	Active 0.71
Diclofenac potassium	98.82%	0.734	0.448	0.986	0.746	0.963	Active 0.79
Ibuprofen	94.37%	0.778	0.538	0.795	0.416	0.982	Inactive
Indomethacin	98.71%	0.959	0.919	0.987	0.773	0.953	Active 0.86
Mefenamic acid	96.28%	1.419	0.932	0.123	0.586	0.401	Active 0.83
Meloxicam	98.38%	0.555	0.977	0.084	0.888	0.971	Active 0.75
Naproxen	96.57%	2.269	0.221	0.975	0.102	0.945	Active 0.65
Naproxen sodium	89.19%	3.881	0.11	0.975	0.078	0.915	Active 0.83

PPB (Plasma Protein Binding): Plasma protein binding rate, referring to the proportion of a drug that binds to plasma proteins in the blood. This parameter affects the free concentration of the drug in the blood, which in turn influences its distribution and action. Drugs with high plasma protein binding rates may lead to insufficient free concentrations in target tissues, potentially affecting efficacy

CL (Clearance): Clearance, referring to the volume of fluid from which a drug is completely removed per unit time, reflecting the rate at which the drug is cleared from the body. A drug’s clearance affects its exposure time in the body

Rat Oral Acute Toxicity: Oral acute toxicity in rats, typically referring to the acute toxic response observed in rats after oral administration of a drug

NR-PPAR-gamma (NR-PPAR-γ): Non-active Peroxisome Proliferator-Activated Receptor gamma. PPAR-γ is a nuclear transcription factor that regulates fatty acid metabolism and insulin sensitivity, influencing metabolic balance

CYP2C9 inhibitor/substrate: CYP2C9 inhibitor/substrate. CYP2C9 is an important drug-metabolizing enzyme involved in the metabolism of many drugs, including some non-steroidal anti-inflammatory drugs (NSAIDs)

Cytochrome CYP2C9: Cytochrome P450 2C9, an important drug-metabolizing enzyme involved in the metabolism of many drugs (such as NSAIDs) Polymorphisms in the CYP2C9 gene may lead to reduced drug metabolism efficiency, potentially affecting drug efficacy and safety

### Identification of core genes

**Fig. 3:**
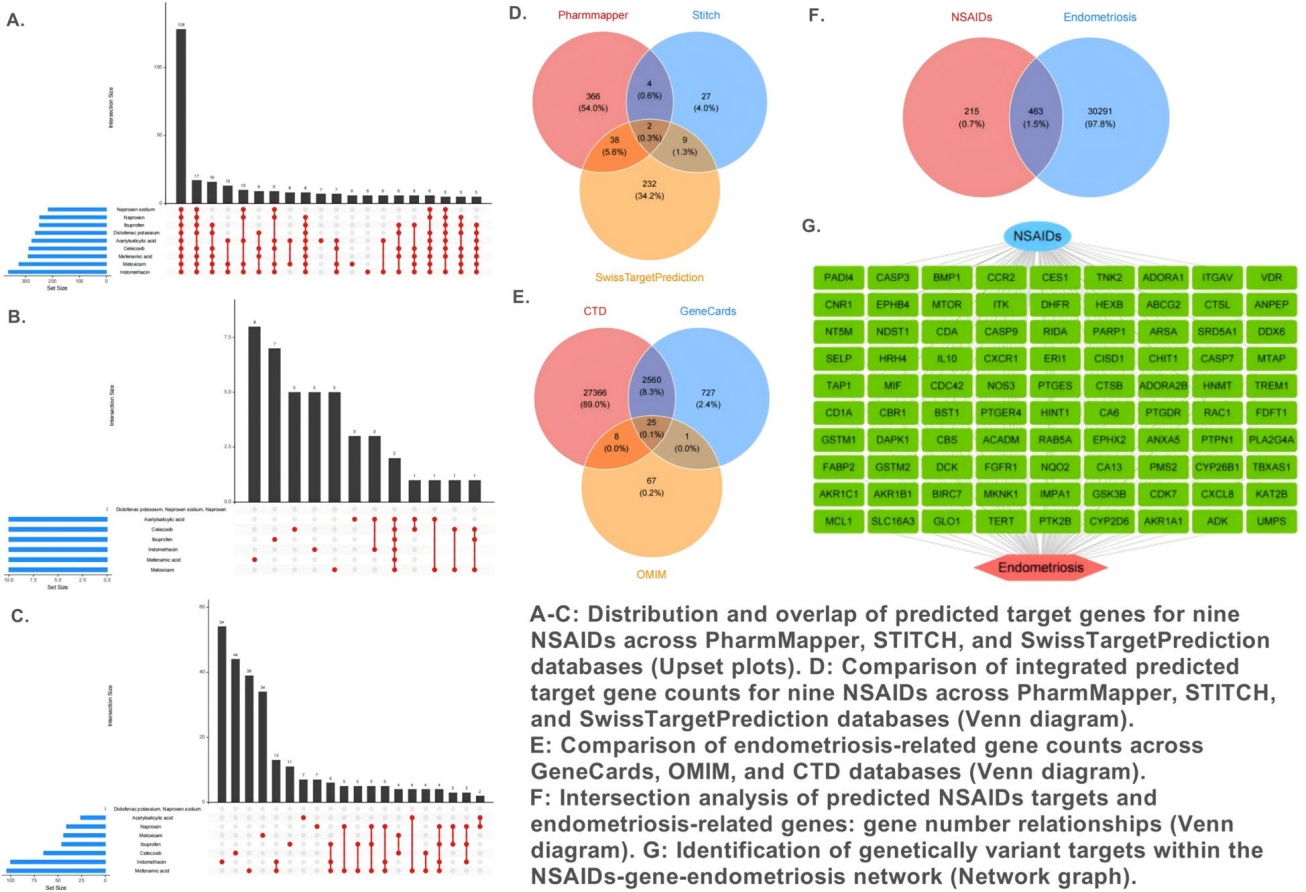
Identification of potential intersecting targets of NSAIDs and endometriosis through multi-omics. In Figures A-C, the horizontal axis of the Upset plot represents the set of target genes predicted for nine NSAIDs in the PharmMapper database, while the vertical axis indicates the number of these genes. Each point represents a gene set, with the horizontal position of the point indicating the number of NSAIDs included in that set, and the vertical height representing the number of genes in that set. The stacking of points visually illustrates the number of shared target genes among different NSAIDs. The matrix and bar chart at the bottom further quantify the gene overlap among different combinations of NSAIDs

As illustrated in Fig. 3, we systematically constructed a molecular association network between non-steroidal anti-inflammatory drugs (NSAIDs) and endometriosis (EMS) by integrating multiple predictive and genetic databases. First, by integrating the predicted targets for nine NSAIDs from the PharmMapper, STITCH, and SwissTargetPrediction databases with EMS-related genes from the GeneCards, OMIM, and CTD databases, we identified a total of 463 overlapping targets (Fig. 3D-F). Subsequently, these targets were screened using genetic databases, which identified 90 candidate targets suitable for use as instrumental variables (IVs) in the Mendelian randomization (MR) analysis (Fig. 3G, Supplementary Table 1). The lists of targets for each of the nine NSAIDs are detailed in Supplementary Tables 2–10. The databases were accessed on May 16, 2023.

#### Results of Mendelian randomization analysis

**Fig. 4:**
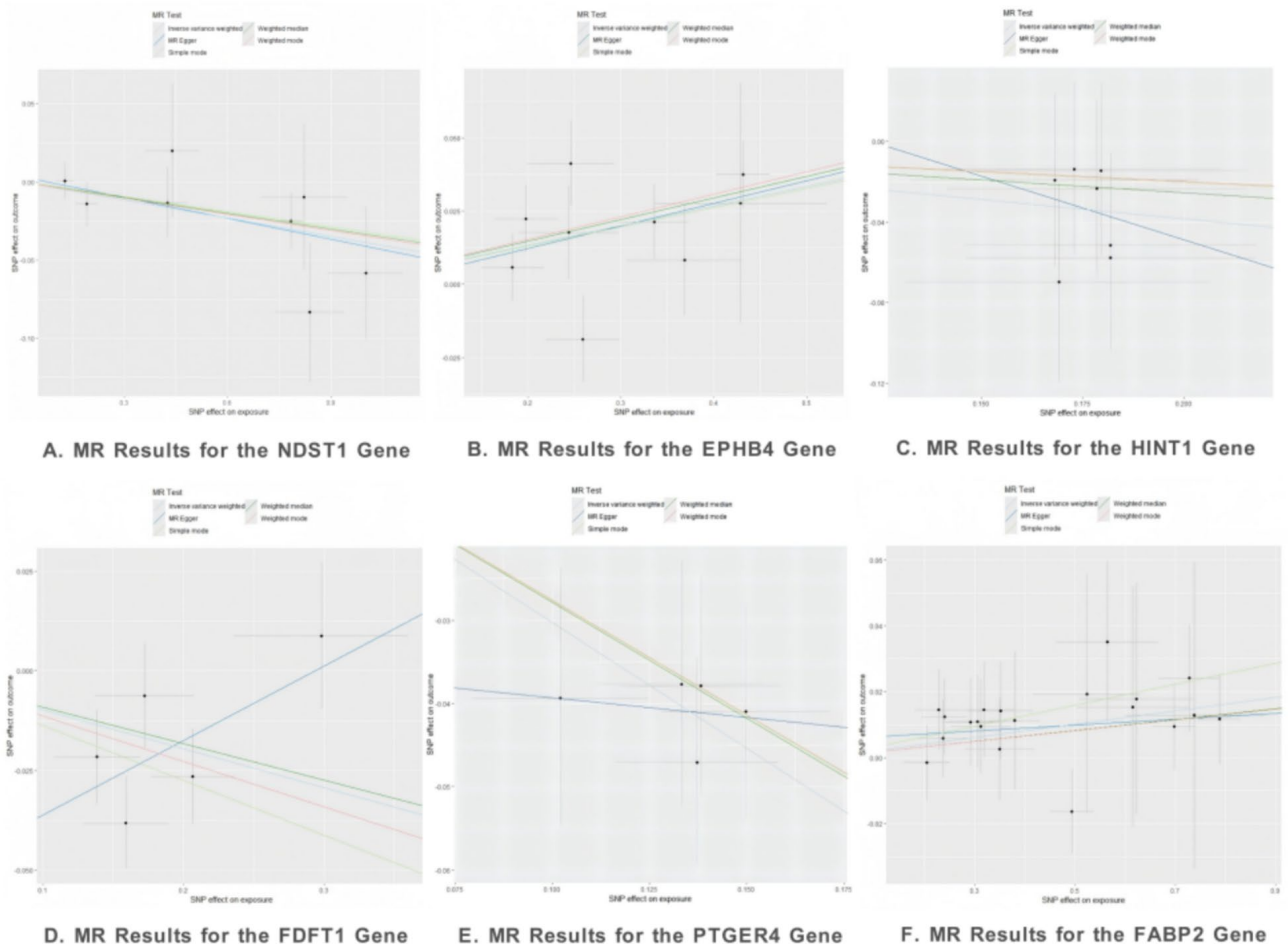
Results of the Mendelian Randomization analysis (In the figure, different colored lines represent five different analytical methods: MR Egger, Weighted Median, Inverse Variance Weighted, Simple Mode, and Weighted Mode.)

We employed Mendelian randomization (MR) analysis to evaluate the potential causal relationships between the target genes of these NSAIDs (as the exposure) and the outcome of endometriosis (EMS) (Fig. 4). All harmonized exposure and outcome data used for the MR analysis, including detailed information on the instrumental variables (SNPs) and their F-statistics (F > 10), are provided in Supplementary Tables 11–22.

As shown in Table 4, after rigorous Bonferroni and FDR correction, the causal associations for *EPHB4* (p_bonferroni = 0.0018, p_fdr = 0.0009) (Supplementary Table 23) and *PTGER4* (p_bonferroni = 1.687E-10, p_fdr = 1.687E-10) (Supplementary Table 28) remained highly significant. For other genes, including *NDST1* (Supplementary Table 27), *HINT1* (Supplementary Table 26), *FDFT1* (Supplementary Table 25), and *FABP2* (Supplementary Table 24), the associations did not reach statistical significance after correction.

**Table 4:** Causal target gene details (IVW Method)

Gene	nSNP	Beta	Se	*P*-value	OR (lci95, uci95)	pval_bonferroni	pval_fdr	Pval_BWMR
*NDST1*	8	−0.039	0.016	0.014	0.96 (0.93–0.99)	0.071	0.071	0.015
*EPHB4*	9	0.067	0.019	0.0004	1.07 (1.03–1.11)	0.0018	0.0009	0.0010
*HINT1*	7	−0.192	0.096	0.046	0.83 (0.68–1.00)	0.228	0.228	0.052
*FDFT1*	5	−0.098	0.047	0.039	0.91 (0.83–1.00)	0.194	0.097	0.014
*PTGER4*	5	−0.302	0.046	3.373E-11	0.74 (0.68–0.81)	1.687E-10	1.687E-10	2.781E-09
*FABP2*	20	0.020	0.008	0.010	1.02 (1.00–1.04)	0.050	0.050	0.011

Gene: The target protein investigated as the exposure in the MR analysis. nSNP: The number of independent Single Nucleotide Polymorphisms (SNPs) used as instrumental variables for the gene. Beta (β): The estimated causal effect size from the IVW method. It represents the change in the log-odds of the outcome (EMS) per unit increase in the genetically predicted expression of the gene. A positive value indicates a risk-increasing effect, while a negative value indicates a protective effect. Se: The standard error of the Beta estimate, indicating the precision of the estimate. *P*-value: The original p-value from the IVW MR analysis, testing the null hypothesis of no causal effect. OR (lci95, uci95): The Odds Ratio and its 95% confidence interval, derived from the Beta value. An OR > 1 suggests a risk effect, and an OR < 1 suggests a protective effect. If the 95% CI does not include 1, the effect is considered statistically significant. MR: Mendelian Randomization. IVW: Inverse Variance Weighted method, a primary MR analysis method that combines the Wald ratio estimates from each SNP into a single causal estimate

Explanations for Multiple Testing and Alternative Method P-values:

pval_bonferroni: The p-value after Bonferroni correction. This is a highly conservative method used to adjust for multiple hypothesis testing (in this case, for 6 genes) and control the family-wise error rate. The significance threshold is divided by the number of tests. A result is considered significant only if its pval_bonferroni is below this new threshold

pval_fdr: The p-value after False Discovery Rate (FDR) correction. This is a less conservative and often more powerful method for multiple testing correction. It controls the expected proportion of false positives among all results declared significant. A common threshold is FDR < 0.05

Pval_BWMR: The *p*-value from the Bayesian Weighted Mendelian Randomization (BWMR) analysis. This is an alternative, independent MR method based on a Bayesian statistical model. It is robust to certain violations of MR assumptions, such as horizontal pleiotropy. Its result serves as a sensitivity analysis to validate the findings from the primary IVW method

Furthermore, the results from the Bayesian Weighted Mendelian Randomization (BWMR) analysis were highly consistent with those from the primary method (IVW). BWMR also confirmed the significant effects for *EPHB4* (P_BWMR = 0.0010) and *PTGER4* (P_BWMR = 2.781E-09). Additionally, the BWMR analysis supported the associations for *NDST1* (P_BWMR = 0.015), *FDFT1* (P_BWMR = 0.014), and *FABP2* (P_BWMR = 0.011), providing additional evidence for these suggestive findings from a Bayesian statistical framework. Taken together, the causal associations of *EPHB4* and *PTGER4* with endometriosis received the most robust support.

#### Sensitivity analyses

To ensure the robustness of our Mendelian randomization (MR) results, we conducted a series of sensitivity analyses to assess potential pleiotropy, heterogeneity, directionality, and colocalization (Table 5; detailed results are presented in Supplementary Tables 23–28). The MR-Egger regression intercept test showed that for the six target genes—*EPHB4*, *PTGER4*, *NDST1*, *HINT1*, *FDFT1*, and *FABP2*—the intercept terms were not significant (*P* > 0.05), indicating no evidence of horizontal pleiotropy. Similarly, Cochran’s Q test revealed no significant heterogeneity (*P* > 0.05), and the MR-PRESSO analysis detected no outliers that required removal. These results collectively support that our primary MR analysis was not significantly confounded by pleiotropy or heterogeneity. Furthermore, the results from the leave-one-out analysis, MR effect forest plots, and funnel plots provided additional support for the robustness of our conclusions (Supplementary Figs. 1–18). Steiger directionality testing indicated that for *EPHB4*, *PTGER4*, *NDST1*, *HINT1*, and *FDFT1*, 100% of the instrumental variables (SNPs) had the correct causal direction, thus statistically ruling out the possibility of reverse causation (Steiger directionality test results are in Supplementary Tables 23–28; detailed reverse MR results are in Supplementary Tables 29–34).

**Table 5:** Results of sensitivity analyses for the causal effects of six NSAID target genes on endometriosis

Gene	MR Egger Intercept (P)	Heterogeneity Q (P)	MR-PRESSO Outliers	Steiger Direction	Reverse MR P-value	Colocalization PP4
*EPHB4*	−0.003 (0.878)	11.5 (0.119)	None	9 SNPs (100%) have the correct direction	/	56.7%
*PTGER4*	−0.035 (0.581)	0.34 (0.952)	None	5 SNPs (100%) have the correct direction	/	60.6%
*NDST1*	0.003 (0.781)	3.02 (0.806)	None	8 SNPs (100%) have the correct direction	/	2.33%
*HINT1*	0.078 (0.894)	1.50 (0.913)	None	7 SNPs (100%) have the correct direction	/	0.005%
*FDFT1*	−0.055 (0.143)	4.17 (0.244)	None	5 SNPs (100%) have the correct direction	/	6.56%
*FABP2*	0.005 (0.496)	7.75 (0.982)	None	20 SNPs (100%) have the correct direction	/	0.973%

Gene: The six target genes of Non-Steroidal Anti-Inflammatory Drugs (NSAIDs) investigated in this study

MR Egger Intercept (P): Test for horizontal pleiotropy. The value in parentheses is the P-value. A P-value > 0.05 suggests the absence of horizontal pleiotropy, indicating that the instrumental variables are not associated with the outcome through pathways other than the exposure, which strengthens the reliability of the MR results

Heterogeneity Q (P): Test for heterogeneity. The value in parentheses is the P-value. A P-value > 0.05 indicates no significant heterogeneity among the causal effect estimates from different instrumental variables, suggesting the results are robust

MR-PRESSO Outliers: Test for outliers. If outliers are detected, they should be removed and the analysis re-performed to obtain more robust causal estimates. “None” in this column indicates that no outliers were identified

Steiger Direction: Directionality test. Used to confirm the direction of causality is from “gene expression → disease” rather than the reverse. For all genes listed, the instrumental variables support the correct direction

Reverse MR P-value: Reverse Mendelian Randomization test. Used to test for the existence of a causal effect from “disease → gene expression”. A “/” indicates that the test could not be performed due to an insufficient number of SNPs

Colocalization PP4: Colocalization analysis. The PP4 value represents the posterior probability that the causal variant for the gene expression and the causal variant for the disease risk are the same variant. A PP4 value > 80% is generally considered strong evidence for colocalization, making the causal association more credible

Colocalization analysis provided crucial evidence for assessing the reliability of the causal associations. The results showed that *PTGER4* (PP4 = 60.6%) and *EPHB4* (PP4 = 56.7%) had relatively high PP4 values, suggesting that their causal associations are likely driven by a shared causal variant. In contrast, the PP4 values for *NDST1*, *HINT1*, *FDFT1*, and *FABP2* were all low, indicating that the association between the exposure and outcome for these genes is more likely due to linkage disequilibrium rather than true colocalization.

Integrating the results from multiple testing correction, Bayesian model validation, and all aforementioned sensitivity analyses, the causal associations of *PTGER4* and *EPHB4* with endometriosis demonstrated the highest robustness and reliability. Therefore, subsequent statistical power assessment and mechanistic investigations will prioritize these two genes.

#### Focus on core genes: statistical power analysis for *PTGER4* and *EPHB4*

To assess the statistical sensitivity of our study design, we calculated the minimum causal effect detectable with 80% power (the Minimum Detectable Effect, MDE). As shown in Table 6, for *PTGER4*, the MDE was 0.8107, whereas the observed odds ratio (OR) was 0.74 (95% CI: 0.68–0.81), which is below this MDE. For *EPHB4*, the MDE was 1.0117, and the observed OR was 1.07 (95% CI: 1.03–1.11), which is above this MDE. Furthermore, the statistical power of our study design to detect the lower limit of the confidence interval, the observed value, and the upper limit of the OR for both genes exceeded 99.9%.

**Table 6:** Statistical power assessment for the causal effects of *PTGER4* and *EPHB4* on endometriosis

Gene	Observed OR (95% CI)	Average R^2^ (%)	Average F-statistic	Minimum Detectable OR (80% Power)	Power to Detect Lower CI OR	Power to Detect Observed OR	Power to Detect Upper CI OR
*PTGER4*	0.74 (0.68–0.81)	0.1346	38.55	0.8107	> 99.9%	> 99.9%	> 99.9%
*EPHB4*	1.07 (1.03–1.11)	0.2087	57.43	1.0117	> 99.9%	> 99.9%	> 99.9%

#### Summary of core findings

By integrating the results from the Mendelian randomization analysis, multiple testing correction, Bayesian model validation, and a series of sensitivity analyses (including tests for pleiotropy, heterogeneity, directionality, and colocalization), we can stratify our findings into two tiers. The causal associations of *PTGER4* and *EPHB4* with endometriosis exhibited the highest robustness (Table 7). The associations for these two targets not only remained significant after rigorous statistical correction but, more critically, were supported by strong genetic evidence from colocalization analysis, indicating that they are likely driven by a shared causal variant (PP4 > 50%). The power assessment further confirmed that our study design had sufficient statistical power to detect the effects of these two genes. In contrast, the associations for *NDST1*, *HINT1*, *FDFT1*, and *FABP2* should be considered suggestive findings. Although they showed statistical associations in some analyses, their low PP4 values from colocalization analysis suggest that these associations are more likely due to linkage disequilibrium rather than true colocalization, rendering the evidence for a causal relationship insufficient.

**Table 7:** Mendelian randomization analysis of nine non-steroidal anti-inflammatory drug (NSAID) target genes and their effects on endometriosis

NSAID	Target Gene(s)	Initial MR Result (P-value)	Final Result After Sensitivity Analyses
Acetylsalicylic acid	*EPHB4, NDST1, HINT1*	Significant (< 0.05)	Robust: *EPHB4*; Not Robust: *NDST1*, *HINT1*
Celecoxib	*EPHB4, NDST1, HINT1*	Significant (< 0.05)	Robust: *EPHB4*; Not Robust: *NDST1*, *HINT1*
Diclofenac potassium	*EPHB4*	Significant (< 0.05)	Robust: *EPHB4*
Indomethacin	*EPHB4, PTGER4, FDFT1, NDST1, HINT1*	Significant (< 0.05)	Robust: *EPHB4*, *PTGER4*; Not Robust: *FDFT1*, *NDST1*, *HINT1*
Ibuprofen	*EPHB4*	Significant (< 0.05)	Robust: *EPHB4*
Mefenamic acid	*EPHB4, FABP2, HINT1*	Significant (< 0.05)	Robust: *EPHB4*; Not Robust: *FABP2*, *HINT1*
Meloxicam	*EPHB4, NDST1, HINT1*	Significant (< 0.05)	Robust: *EPHB4*; Not Robust: *NDST1*, *HINT1*
Naproxen	*EPHB4, NDST1, HINT1, FDFT1*	Significant (< 0.05)	Robust: *EPHB4*; Not Robust: *NDST1*, *HINT1*, *FDFT1*
Naproxen sodium	*EPHB4, NDST1, HINT1*	Significant (< 0.05)	Robust: *EPHB4*; Not Robust: *NDST1*, *HINT1*

Therefore, subsequent mechanistic investigations will prioritize *PTGER4* and *EPHB4*, for which the evidence is most conclusive. We will employ functional enrichment analysis to gain an in-depth understanding of their potential pathogenic mechanisms from a biological functional perspective. Furthermore, we will use molecular docking simulations to explore their interaction patterns with relevant drug molecules at the atomic level, thereby providing multi-dimensional biological evidence to support the causal associations discovered through MR.

### Functional enrichment analysis of core genes based on drug targeting

To further investigate the potential pathogenic mechanisms of *PTGER4* and *EPHB4* in endometriosis and to elucidate the targets of relevant non-steroidal anti-inflammatory drugs (NSAIDs), we performed a functional enrichment analysis on these core genes based on drug-target interactions. The results from the combined network toxicology and Mendelian randomization analysis revealed that eight NSAIDs (Celecoxib, Diclofenac potassium, Indomethacin, Ibuprofen, Mefenamic acid, Meloxicam, Naproxen and Naproxen sodium) all target *EPHB4*. In contrast, indomethacin targets both *PTGER4* and *EPHB4*. Based on this unique targeting pattern, we present the results of the enrichment analysis in two groups: (1) pathways co-regulated by NSAIDs that target *EPHB4*; and (2) unique pathways potentially regulated by indomethacin through both *PTGER4* and *EPHB4*.

#### Pathways co-enriched by NSAIDs targeting *EPHB4*

Acknowledging the limitations of pathway enrichment analysis based on a single gene, we utilized the STRING database to construct an interaction network comprising *EPHB4* and its upstream and downstream related genes, which included EFNA5, EFNB3, EFNB2, ABL1, *EPHB4*, EFNB1, RASA1, and NGEF. KEGG enrichment analysis of these network genes revealed that the “Axon guidance” pathway was the most significantly enriched (p.adjust = 3.86e-13), with all 8 genes in the network participating in this pathway. Additionally, the “Ras signaling pathway” was also significantly enriched (p.adjust = 6.30e-03).

To further investigate the specific biological functions of *EPHB4*, we performed Gene Ontology (GO) enrichment analysis. At the Biological Process (BP) level, “ephrin receptor signaling pathway” was the most significantly enriched term (p.adjust = 3.84e-15), directly highlighting the core function of *EPHB4*. Concurrently, processes highly related to axon guidance, such as “axonogenesis” and “axon development,” were also significantly enriched, corroborating the KEGG results. Notably, *EPHB4* was also involved in several key processes directly related to cell migration and angiogenesis, such as “cell migration involved in sprouting angiogenesis” and “endothelial cell migration,” which are closely linked to the vascularization and invasive growth characteristics of endometriotic lesions. At the Molecular Function (MF) level, the results further refined the mode of action of the *EPHB4* network. We found that the core molecular functions of this network were highly enriched, with “protein tyrosine kinase activity” (p.adjust = 3.86e-04) and “ephrin receptor activity” (p.adjust = 3.86e-04) being the most significant. This suggests that *EPHB4*, along with its ligand EFNB3 and downstream kinase ABL1, forms a functional complex that efficiently transmits signals by catalyzing tyrosine phosphorylation. This finding not only confirms the molecular mechanism of *EPHB4* as a membrane-bound receptor tyrosine kinase but also emphasizes its central role in the network, acting in concert with key partners.

**Fig. 5:**
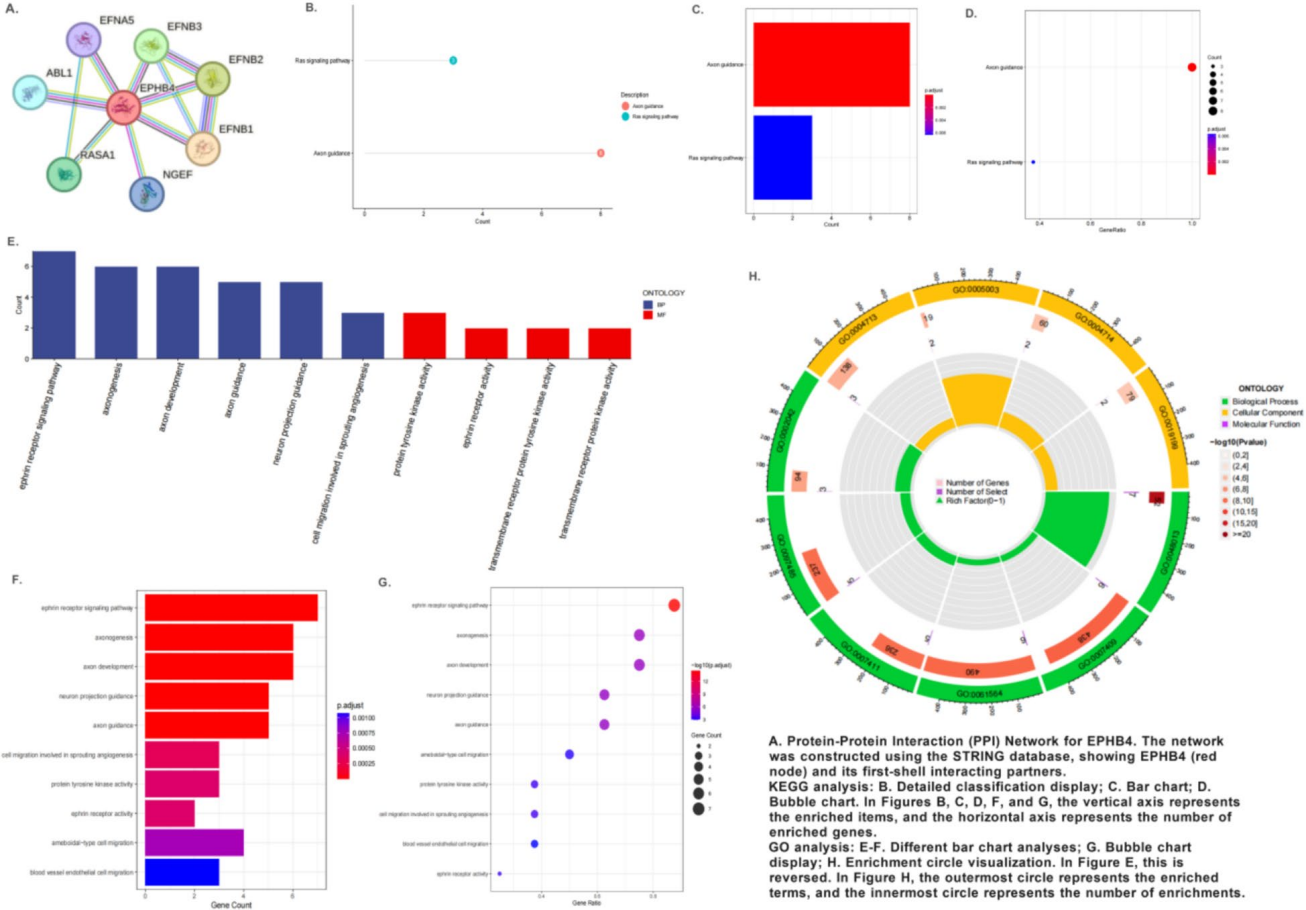
*EPHB4* interaction network and functional enrichment analysis

Taken together, these enrichment analyses collectively depict a clear picture: *EPHB4*, acting as a key membrane receptor, regulates downstream cell migration and angiogenesis processes by activating the ephrin signaling pathway, thereby playing a “risk” role in the pathogenesis of endometriosis. This leads to a bold hypothesis: in addition to alleviating symptoms through the classic anti-inflammatory pathway, the eight NSAIDs widely used to treat endometriosis may also inadvertently promote cell migration and angiogenesis related to disease progression by activating *EPHB4*—a risk gene—and its downstream pathways, thereby potentially exacerbating the disease’s pathological process. This finding poses a novel challenge to the safety of NSAIDs in the treatment of endometriosis. The interaction networks and enrichment analysis results are detailed in Fig. 5, and the detailed data for KEGG and GO enrichment are provided in Supplementary Tables 35 and 36, respectively.

#### Unique enriched pathways of indomethacin

Unlike other NSAIDs, indomethacin exhibits a unique enrichment profile due to its dual targeting of both *PTGER4* and *EPHB4*. To investigate the potential synergistic biological effects of this dual-targeting property, we performed a functional enrichment analysis using *PTGER4* and *EPHB4* as the core gene set. Given that the analysis included only two genes, we moderately relaxed the screening criteria for the KEGG analysis (p.adjust < 0.25) to more comprehensively capture potential biological signals, while maintaining a stricter standard for the GO analysis (p.adjust < 0.05).

The KEGG pathway enrichment analysis revealed the broad scope of indomethacin’s action. The significantly enriched pathways not only included “Axon guidance” (contributed by *EPHB4*), which we had previously focused on, but also encompassed several key physiological processes contributed by *PTGER4*, such as “Renin secretion,” “Inflammatory mediator regulation of TRP channels,” and “Efferocytosis.” This suggests that indomethacin may exert its therapeutic effects by simultaneously influencing multiple dimensions, including nerve development, vascular regulation, inflammatory perception, and cell clearance.

The GO enrichment analysis further focused the molecular mechanism of indomethacin on the core functions of *PTGER4*. At the Biological Process (BP) level, the most significantly enriched terms were directly related to the cellular response to prostaglandin E (PGE), such as “cellular response to prostaglandin E stimulus” (p.adjust = 4.16e-02) and “response to prostaglandin,” which precisely reflects the pharmacological properties of indomethacin as a ligand for *PTGER4*. Additionally, a series of terms related to immune cell migration, activation, and differentiation (e.g., “eosinophil migration,” “T cell differentiation”) and the regulation of inflammatory response (e.g., “positive/negative regulation of inflammatory response”) were also significantly enriched, which is highly consistent with the potent anti-inflammatory activity of indomethacin. Notably, *EPHB4*-related terms such as “ephrin receptor signaling pathway” and “cell migration” also appeared in the enrichment results, once again confirming its dual-targeting characteristic. At the Molecular Function (MF) level, the results clearly pointed to the molecular targets of indomethacin. “Prostaglandin receptor activity” and “ephrin receptor activity” were simultaneously enriched, providing the most direct molecular-level evidence that indomethacin acts on both *PTGER4* and *EPHB4*.

**Fig. 6:**
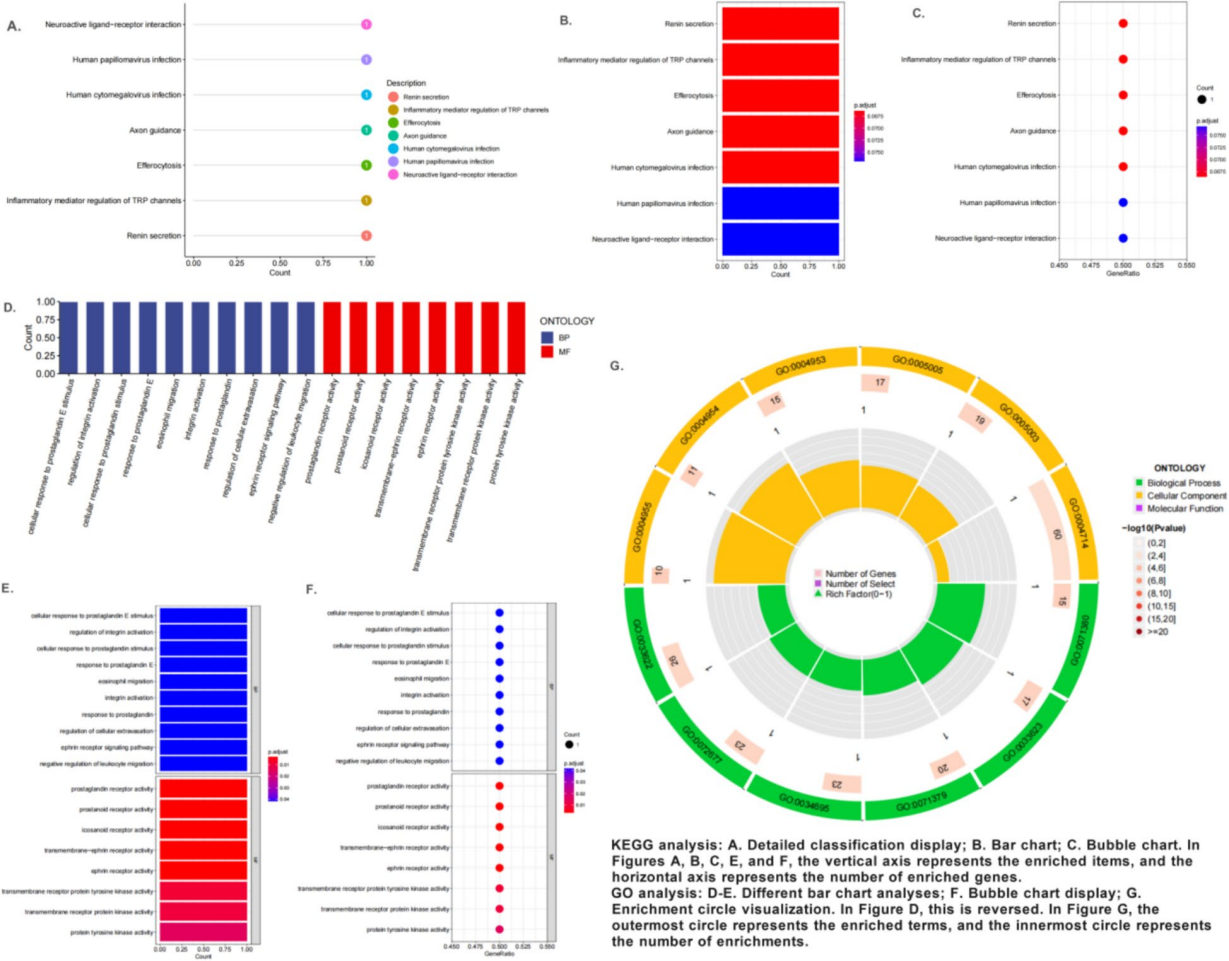
The dual mechanism of indomethacin: enrichment analysis based on *PTGER4* and *EPHB4*

In summary, the uniqueness of indomethacin lies in its potential to play a dual role as both a “healer” and a “promoter.” It exerts its known anti-inflammatory therapeutic effects through the *PTGER4* pathway but may inadvertently activate mechanisms that promote disease progression through the *EPHB4* pathway. This interplay between “risk and protection” profoundly reveals the complexity of NSAID action in the treatment of endometriosis and provides a novel molecular-level explanation for the clinical phenomena of poor efficacy or recurrent disease in some patients. These enrichment analysis results are detailed in Fig. 6, and the detailed data for KEGG and GO enrichment are provided in Supplementary Tables 37 and 38.

### Molecular docking results

**Fig. 7:**
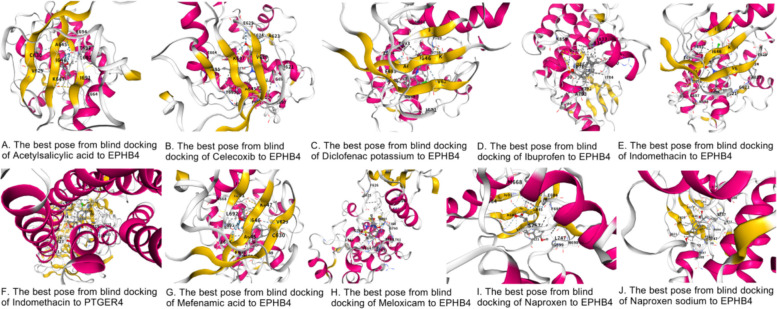
Molecular docking modes of NSAIDs with key target proteins

To evaluate the molecular interactions between the core genes identified by Mendelian randomization (MR) analysis and non-steroidal anti-inflammatory drugs (NSAIDs), we performed molecular docking experiments (detailed results in Fig. 7, Table 8). To ensure the accuracy and biological relevance of the docking, we rigorously screened the three-dimensional (3D) structures of the core targets. For the risk gene *EPHB4*, we selected a high-resolution crystal structure of its kinase domain in complex with an inhibitor (PDB ID: 6FNM, 1.157 Å), which provided the most precise atomic coordinates and a reliable reference binding pocket for docking. For the protective gene *PTGER4*, a G-protein coupled receptor (GPCR), structural determination is more challenging. After evaluating multiple structures, we excluded some that, despite high resolution, were artificial chimeras or contained mutations. Ultimately, we chose a native, complete human *PTGER4* structure in complex with an antagonist (PDB ID: 5YWY, 3.2 Å) to ensure the simulation results could genuinely reflect drug-target interactions under physiological conditions. The structural and sequence data used in this study were retrieved from the PDB and UniProt databases on October 24, 2025.

**Table 8:** Molecular docking results of multiple non-steroidal anti-inflammatory drugs with their causal targets

Ligand	Target Gene	Pocket ID	Binding Affinity (kcal/mol)	Cavity Volume (Å^3^)
Indomethacin	*PTGER4*	C2	−9.0	2207
Mefenamic acid	*EPHB4*	C1	−8.9	496
Celecoxib	*EPHB4*	C1	−8.8	496
Indomethacin	*EPHB4*	C1	−8.4	496
Meloxicam	*EPHB4*	C3	−7.7	359
Diclofenac potassium	*EPHB4*	C1	−7.4	496
Naproxen	*EPHB4*	C1	−7.2	496
Naproxen sodium	*EPHB4*	C1	−7.0	496
Acetylsalicylic acid	*EPHB4*	C1	−6.2	496
Ibuprofen	*EPHB4*	C4	−6.2	351

Vina score (kJ/mol): Vina is a software used for molecular docking, which can predict the binding affinity between small molecules (such as drugs, endocrine disruptors, etc.) and biomacromolecules (such as proteins). The Vina score indicates the strength of this binding, typically expressed in energy units of kilojoules per mole (kJ/mol). The lower the score, the more stable the binding

Cavity volume (Å3): In molecular biology, a cavity usually refers to the space inside or on the surface of macromolecules like proteins, which can accommodate small molecules such as drugs or ligands. The cavity volume is measured in cubic angstroms (Å3), representing the size of this space. This parameter is important for understanding molecular docking and binding characteristics

The docking results revealed several key findings. As shown in Table 8, all tested NSAIDs could form stable complexes with either *EPHB4* or *PTGER4*, with binding affinities ranging from −6.2 to −9.0 kcal/mol, providing direct atomic-level evidence for our MR analysis. Notably, indomethacin demonstrated the strongest binding potential. It not only exhibited a very strong binding affinity with the *EPHB4* protein (−8.4 kcal/mol) but also showed a remarkable binding affinity of −9.0 kcal/mol with our other robust target, *PTGER4*, representing the best result among all combinations. This finding provides a powerful explanation for why indomethacin exhibited such a significant genetic effect in the MR analysis. Furthermore, mefenamic acid and celecoxib also showed binding affinities with *EPHB4* below −8.8 kcal/mol, indicating that they are also potential potent inhibitors of *EPHB4*. Interestingly, we observed that multiple drugs (including indomethacin, mefenamic acid, celecoxib, diclofenac potassium, naproxen, and its sodium salt) could all bind to the same key pocket (C1) on *EPHB4*, which has a volume of 496 Å^3^, suggesting that this may be a druggable “hotspot” region.

In summary, these computational results not only validate the interactions between NSAIDs and the *EPHB4*/*PTGER4* targets but also elucidate the potential differences in the magnitude of drug effects at the molecular level, providing clear priorities and a structural basis for subsequent experimental validation and drug optimization.

## Discussion

The safety of non-steroidal anti-inflammatory drugs (NSAIDs) in the treatment of endometriosis is a long-standing yet unresolved critical clinical issue. By integrating multi-omics analysis with structural biology simulations, this study provides the first systematic answer to this question from the perspectives of causal inference and the atomic level. Our core finding is that the effects of NSAIDs on EMS are not uniform but rather exhibit target-oriented heterogeneity. We identified *EPHB4* as a central hub mediating the potential “risk-promoting” effects of multiple NSAIDs, while simultaneously revealing *PTGER4* as the key to indomethacin’s unique “risk-protection trade-off” mechanism. This achievement not only provides a molecular-level basis for decision-making in the precise and individualized application of NSAIDs but also establishes a new research paradigm for drug safety assessment in EMS.

### From a “Homogeneous Class” to “Heterogeneous Agents”: unveiling the target-oriented effects of NSAIDs

**Fig. 8:**
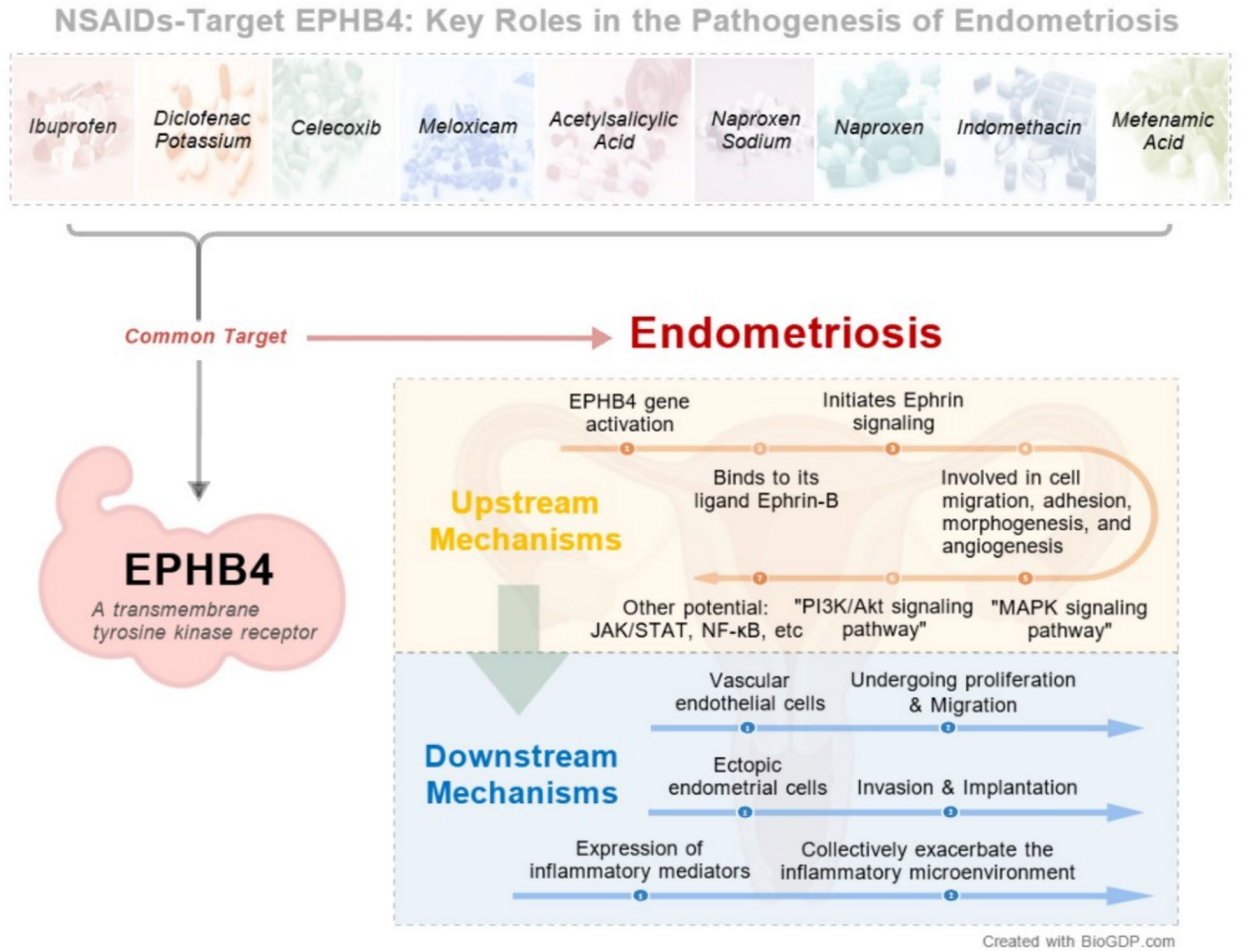
Multiple roles of the core gene *EPHB4*

Previous research has treated NSAIDs as a homogeneous drug class, broadly discussing their risks, which has led to long-standing contradictions in the clinical evidence regarding their safety [74, 75]. Our study, through Mendelian randomization (MR) analysis, fundamentally subverts this traditional view by demonstrating for the first time at the population level that different NSAIDs have essentially different impacts on EMS risk. Based on their target genes, the nine NSAIDs can be clearly divided into two categories, playing distinctly different roles:

Category 1: The “Risk Promoters” Centered on *EPHB4*. Our research found that eight NSAIDs, represented by aspirin and celecoxib, share a common target, the *EPHB4* gene, which has a robust causal association with an increased risk of endometriosis (EMS). Functional enrichment analysis suggests that the pathogenic mechanism of *EPHB4* may primarily revolve around the alteration of cellular behavior. First, by activating the “ephrin receptor signaling pathway,” it theoretically directly regulates cell migration, invasion, and angiogenesis—processes that are central to the formation and development of EMS lesions [76–79]. Second, our bioinformatics analysis further revealed that *EPHB4* acts not only as a “direct driver” of cell invasion by regulating cytoskeletal dynamics but also indirectly remodels the extracellular matrix by participating in heparan sulfate biosynthesis, thereby “paving the way” for cell migration [76, 80]. Therefore, this computational evidence collectively leads to a key hypothesis: while exerting their anti-inflammatory effects, these NSAIDs may inadvertently “step on the gas,” constituting a potential risk of promoting EMS onset and progression, primarily by interfering with the multifunctional gene *EPHB4* across dimensions such as cell migration, invasion, and microenvironment remodeling [76, 80–82] (Fig. 8).

Category 2: The “Risk-Protection Interactors” Represented by Indomethacin. Indomethacin stands as a unique exception in our study. Our analysis revealed that it simultaneously targets *PTGER4* (protective effect, OR < 1) and *EPHB4* (risk effect, OR > 1). This targeting characteristic unveils its dual mechanism of action: on one hand, it exerts a classic anti-inflammatory “protective” effect through the *PTGER4* pathway; on the other hand, as just discussed, it plays a “risk-promoting” role via the *EPHB4* pathway. The complexity of the issue is compounded by the fact that *EPHB4* itself is a functionally diverse key node. Previous studies have shown that *EPHB4* not only regulates cell migration and angiogenesis but also engages in direct physical interactions with other important signaling receptors (such as the insulin receptor), thereby profoundly influencing the stability of the cellular signaling network [82]. This suggests that the impact of indomethacin’s intervention on *EPHB4* may extend far beyond the pathways we have observed. Therefore, indomethacin’s simultaneous action on *PTGER4* and *EPHB4*, this “dual-target” characteristic itself, constitutes the complexity of its effects, offering a potential molecular explanation based on multi-target interactions for the phenomenon of varied patient responses to the drug [83].

### From “Genetic Association” to “Atomic Evidence”: providing a structural basis for heterogeneous effects

To validate the aforementioned genetic findings, we conducted molecular docking experiments, providing direct atomic-level evidence for the interactions between NSAIDs and their target genes. The docking results were highly consistent with our hypothesis. First, indomethacin demonstrated extremely strong binding affinities with both *PTGER4* and *EPHB4* (−9.0 and −8.4 kcal/mol, respectively), structurally explaining the physical basis of its “dual role.” Second, “risk promoters” such as mefenamic acid and celecoxib also exhibited very strong binding affinities with *EPHB4* (< −8.8 kcal/mol). More interestingly, we found that multiple drugs, including indomethacin, could all bind to the same key pocket (C1) on *EPHB4*, strongly suggesting that this pocket is the structural basis mediating the “off-target” risk effects.

### From “Clinical Controversy” to a “New Paradigm”: reshaping the cognitive framework for drug safety assessment in EMS

Based on the above findings, we formally propose the new paradigm of “target-oriented heterogeneous effects.” This paradigm not only provides a unified explanatory framework for previously contradictory clinical evidence but, more importantly, shifts the clinical focus from the vague question of “whether to use NSAIDs” to the core proposition of precision medicine: “how to select safer NSAIDs based on molecular targets.” This paradigm shift is enabled by a triple breakthrough in our study at the methodological, discovery, and value levels:

First, at the methodological level, we innovatively applied a “computationally driven causal inference” strategy to address the long-standing challenge of controversies in NSAID clinical use. Although network toxicology and Mendelian randomization (MR) are existing tools, previous research was either limited to a single method or failed to systematically integrate them to clarify the complex relationships between drugs and diseases. Our study, for the first time, tightly integrates these two approaches, constructing a complete evidence chain from “drug-target” to “target-disease.” This provides a replicable analytical framework for assessing the safety of drugs with complex mechanisms of action and contradictory clinical evidence, such as NSAIDs.

Second, at the discovery level, we unveiled the disruptive concept of “target-oriented heterogeneity.” This study is the first to demonstrate, at the population level through MR analysis, that different NSAIDs have fundamentally different impacts on EMS risk. We not only identified *EPHB4* as a central hub mediating the potential pathogenic effects of multiple NSAIDs but also discovered the unique “dual regulation” phenomenon presented by indomethacin through its simultaneous action on *PTGER4* (protective) and *EPHB4* (risk). This finding advances the research perspective from the “drug class” level to the precise level of “specific drug-specific target,” representing a cognitive leap in the field.

Finally, at the value level, we provide a completely new explanatory framework to resolve long-standing clinical controversies. Our theory of “target-oriented heterogeneity” powerfully explains why previous observational studies have yielded contradictory results. This not only resolves a major controversy within the field but, more importantly, provides a clear target for the development of novel COX inhibitors that avoid activating the *EPHB4* pathway, paving a new avenue for precision pain management in EMS.

### Maximizing benefits and minimizing harms: clinical practice implications under the new paradigm

The new paradigm of “target-oriented heterogeneous effects” revealed in this study provides crucial insights for the clinical management strategy of endometriosis. Currently, a significant disconnect exists between the clinical practice of using NSAIDs as first-line analgesics and the scarcity of high-quality evidence-based medicine. This is reflected not only in their unclear analgesic efficacy but also in the lack of evidence suggesting any single NSAID is superior to another [84–88]. In light of this, our study supports repositioning NSAIDs from a traditional “first-line, long-term pain control tool” to a “short-term, emergency bridge medication,” and establishing an individualized medication principle centered on risk–benefit assessment. This is of paramount importance for protecting the long-term reproductive health of women of childbearing age.

Based on this, drug selection should transcend empirical practice and move towards decision-making based on potential risk profiles. Data from our study provides a direct basis for this decision: among the nine NSAIDs evaluated, eight target only the risk gene *EPHB4*, while indomethacin is the sole drug that targets both the protective gene *PTGER4* and the risk gene *EPHB4*. Despite the complexity of its “risk-protection trade-off,” our MR analysis shows that the protective effect of *PTGER4* (OR = 0.74) is significantly stronger in magnitude than the risk effect of *EPHB4* (OR = 1.07). From a data-driven perspective, this suggests that the overall risk–benefit ratio of indomethacin may be superior to other NSAIDs that only activate the *EPHB4* pathway. Therefore, when NSAIDs are necessary for pain management, indomethacin could be considered a relatively preferable option. Of course, the potential long-term effects of NSAIDs that may interfere with key reproductive signaling pathways (such as those mediated by *EPHB4*) on the ovarian microenvironment and embryo implantation potential must be re-evaluated with caution.

Secondly, optimizing combination therapy strategies is key to mitigating the risks of monotherapy. Combining NSAIDs with hormonal drugs (e.g., progestins, GnRH-a) can not only enhance analgesic efficacy but also effectively reduce reliance on NSAIDs, thereby lowering their potential inhibitory effects on ovulation, luteal function, and follicular development [89–91]. This reduction in reliance is now particularly urgent, as our research reveals a stark possibility: the eight widely used NSAIDs may inadvertently promote cell migration and angiogenesis related to disease progression by activating the risk gene *EPHB4* and its downstream pathways, thereby exacerbating the pathological process. This finding poses a new challenge to the safety of NSAIDs and transforms optimizing combination therapy from an “option” into a “necessity.”

Furthermore, active exploration of novel non-hormonal drugs as alternatives or supplements should be pursued to provide safer options for patients seeking to preserve their fertility [92, 93]. Our study not only provides data-driven support for the rationale of exploring anti-angiogenic drugs but, more critically, suggests that *EPHB4* could serve as a core candidate target for this strategy, thus proposing a new hypothesis for subsequent targeted drug development. Specifically, the enrichment analysis clearly delineates a complete picture of *EPHB4* regulating “sprouting angiogenesis” and “endothelial cell migration” via the “ephrin receptor signaling pathway,” which directly validates the scientific value of an anti-angiogenic therapeutic strategy. Therefore, this study focuses the broad strategy precisely on the core target of *EPHB4*, pointing the way for the development of novel non-hormonal drugs that inhibit this gene or its downstream pathways (such as the Ras signaling pathway), promising to offer safer and more precise interventions for EMS treatment.

Until the advent of future precision medicines, the aforementioned findings also demand corresponding changes in clinical practice. A dynamic monitoring system covering reproductive and metabolic endocrinology must be established to regularly assess the ovarian reserve and metabolic indicators of patients on long-term medication, and to adjust treatment plans accordingly. This requires clinicians to adopt a more prudent strategy when weighing the immediate benefits against the long-term risks of NSAIDs, thereby truly achieving precision medicine for EMS patients that maximizes benefits and minimizes harms while protecting fertility.

In conclusion, this study points out an evolutionary path for EMS treatment strategies, moving from “broad empirical medication” to “precise, targeted intervention.” Through in-depth exploration of core targets such as *EPHB4*, we not only provide a more scientific basis for clinical decision-making but also lay the foundation for the development of next-generation, safer, and more effective targeted drugs.

### Study limitations and future research directions

First, the strength of the causal inference must be cautiously defined. Although Mendelian randomization (MR) provides a powerful tool for assessing potential causal associations, its conclusions are probabilistic in nature, and horizontal pleiotropy remains a potential confounding factor [50]. Therefore, the “causal associations” proposed in this study should be regarded as a strong hypothesis that urgently requires experimental validation. It is worth noting that our findings are supported by multi-level, independent evidence: clinical studies have confirmed that *EPHB4* is highly expressed in ectopic lesions and is strongly associated with VEGFR2 [94]; functional studies have shown that inhibiting *PTGER4* suppresses cell invasion [95], while inhibiting *EPHB4* inhibits lesion growth in mouse models [96]. This body of evidence collectively supports the new paradigm of “target-oriented heterogeneous effects.”

Second, the validation from “target importance” to “drug mechanism of action” still requires direct experimental evidence. Future research should aim to directly validate the core hypotheses in vitro (e.g., endometrial stromal cells, organoids) and in vivo (e.g., EMS mouse models). Specifically, the effects on cell migration, invasion, and angiogenesis can be observed by treating with specific NSAIDs or modulating the expression of *EPHB4* and *PTGER4* using gene-editing techniques [97–99]. In particular, the “risk-protection trade-off” mechanism of indomethacin, proposed for the first time in this study, urgently needs to be validated in these models.

Third, this study is limited by the quality and granularity of public databases [50, 100–102]. To overcome this limitation, future work requires the construction of large-scale, prospective genetic cohorts that include detailed medication histories, EMS subtypes, and fertility outcomes. This would support more fine-grained subgroup MR analyses and enable the development of novel statistical models that incorporate drug-gene-environment interactions to precisely dissect the heterogeneous effects of NSAIDs.

Fourth, the granularity of our population-level analysis limits its ability to directly guide individualized clinical application. Therefore, conducting prospective real-world studies is an essential pathway to bridge this discovery with clinical practice [103]. We recommend designing multi-center prospective cohort studies to systematically collect NSAID usage data and follow up on pain symptoms, disease progression, and fertility indicators, with the goal of building individualized risk–benefit prediction models [104].

Furthermore, to translate genetic findings into clinical tools, we propose the development of a next-generation Genetic Risk Score (GRS) model based on core causal genes such as *PTGER4* and *EPHB4*. Unlike traditional “statistical” GRS models, this model would be strictly limited to causal genes identified through fine-mapping methods, thereby enhancing predictive power and biological interpretability and providing a core tool for achieving “genotype-guided personalized medication” [105].

Finally, to translate foundational discoveries into clinical practice, future work should focus on drug optimization and intervention trials. On one hand, based on the key pocket (C1) of *EPHB4*, computer-aided drug design can be used to screen for or synthesize novel selective COX inhibitors that avoid activating this pathway [106]. On the other hand, pragmatic clinical trials can be designed to compare the advantages and disadvantages of a “precision medication strategy” versus “routine empirical medication” in terms of symptom control and fertility preservation, providing high-level evidence to update clinical guidelines [107].

In conclusion, this study not only reveals the complex role of NSAIDs in EMS but, more importantly, charts a future research roadmap for the field, from basic mechanisms to clinical translation. We anticipate that work in these directions will continue to advance, ultimately bringing safer and more effective treatment options to patients with EMS.

## Conclusion

By integrating network toxicology for target screening, Mendelian randomization for causal inference, enrichment analysis for pathway elucidation, and finally, molecular docking for atomic-level validation, this study is the first to systematically reveal the “target-oriented heterogeneous effects” of non-steroidal anti-inflammatory drugs (NSAIDs) in endometriosis. We identified *EPHB4* as the central hub mediating the potential risk effects of nearly all NSAIDs, while indomethacin emerged as a key exception due to its unique dual targeting of the protective gene *PTGER4* and the risk gene *EPHB4*. This finding elevates the “double-edged sword” attribute of NSAIDs from a vague concept to a precise molecular mechanism. It lays the scientific foundation for a strategic shift in endometriosis treatment from “empirical medication” to “precision-based selection guided by molecular mechanisms,” providing a critical basis for clinical decisions that seek an optimal balance between risk and benefit.

## Supplementary Information

Supplementary Material 1.

Supplementary Material 2.

Supplementary Material 3.

Supplementary Material 4.

Supplementary Material 5.

Supplementary Material 6.

Supplementary Material 7.

Supplementary Material 8.

Supplementary Material 9.

Supplementary Material 10.

Supplementary Material 11.

Supplementary Material 12.

Supplementary Material 13.

Supplementary Material 14.

Supplementary Material 15.

Supplementary Material 16.

Supplementary Material 17.

Supplementary Material 18.

Supplementary Material 19.

Supplementary Material 20.

Supplementary Material 21.

Supplementary Material 22.

Supplementary Material 23.

Supplementary Material 24.

Supplementary Material 25.

Supplementary Material 26.

Supplementary Material 27.

Supplementary Material 28.

Supplementary Material 29.

Supplementary Material 30.

Supplementary Material 31.

Supplementary Material 32.

Supplementary Material 33.

Supplementary Material 34.

Supplementary Material 35.

Supplementary Material 36.

Supplementary Material 37.

Supplementary Material 38.

Supplementary Material 39.

Supplementary Material 40.

Supplementary Material 41.

## Data Availability

The data underlying this article are available in the article and in its online supplementary material.
