# Supplementary material for: The "double-edged sword" effect of non-steroidal anti-inflammatory drugs (NSAIDs) in the treatment of endometriosis (EMS)
Source: Reprod Biol Endocrinol. 2025 Dec 6;24:9. doi: 10.1186/s12958-025-01508-7 (PMC12829210; doi:10.1186/s12958-025-01508-7)

Gene 1: EPHB4

Supplementary Figure 1. Leave-one-out analysis for the Mendelian randomization of EPHB4.


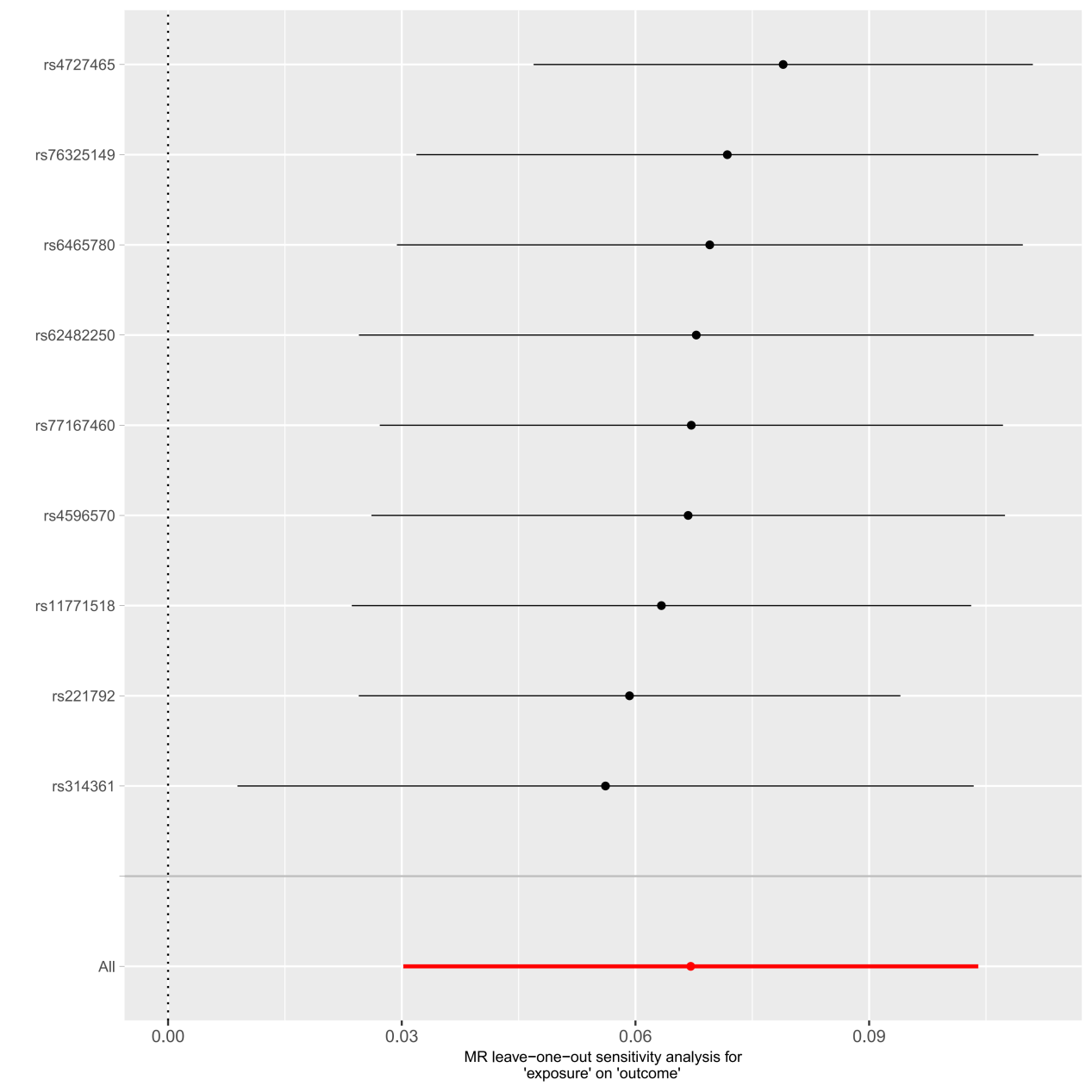


Supplementary Figure 2. Forest plot of the Mendelian randomization estimates for EPHB4.


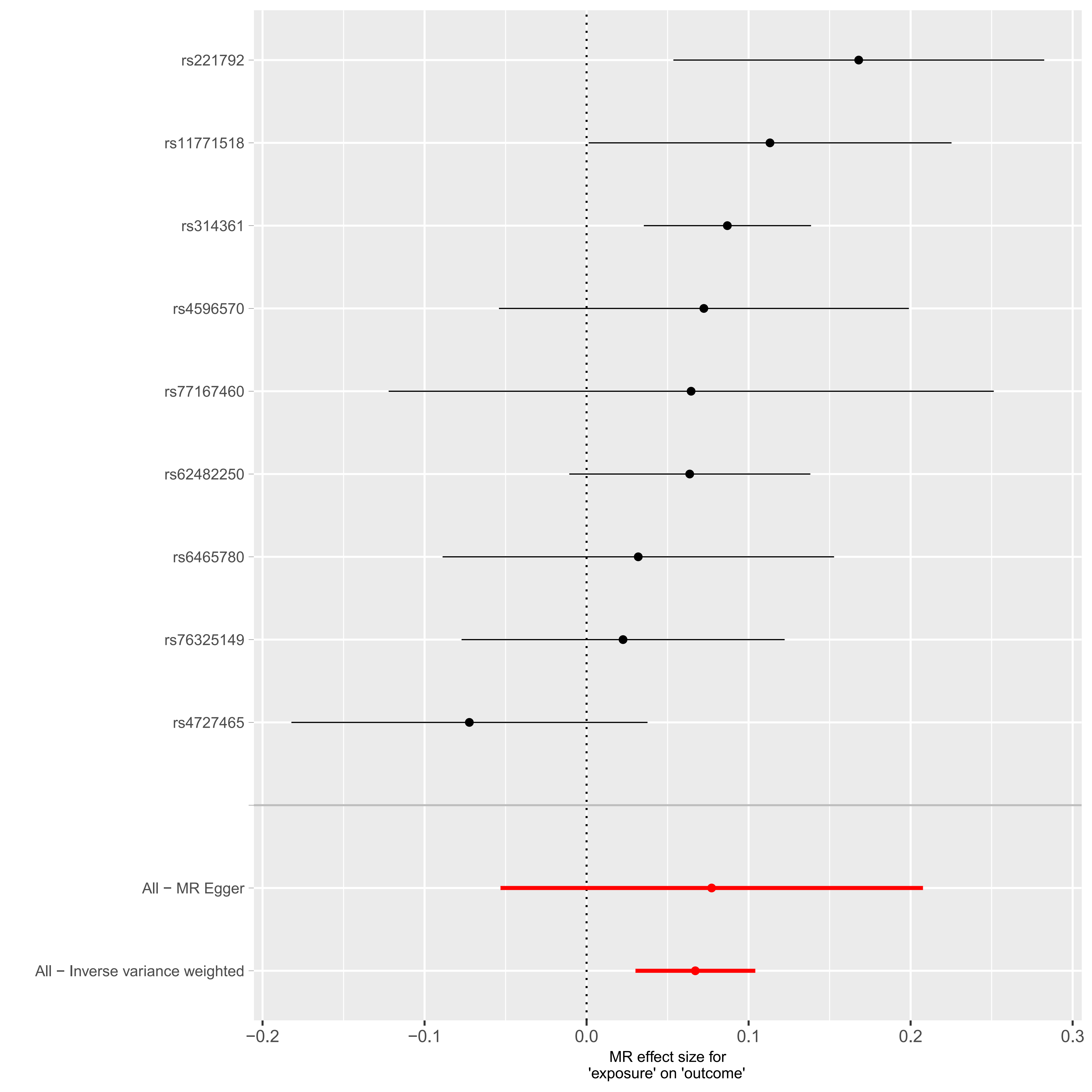


Supplementary Figure 3. Funnel plot for the Mendelian randomization of EPHB4.


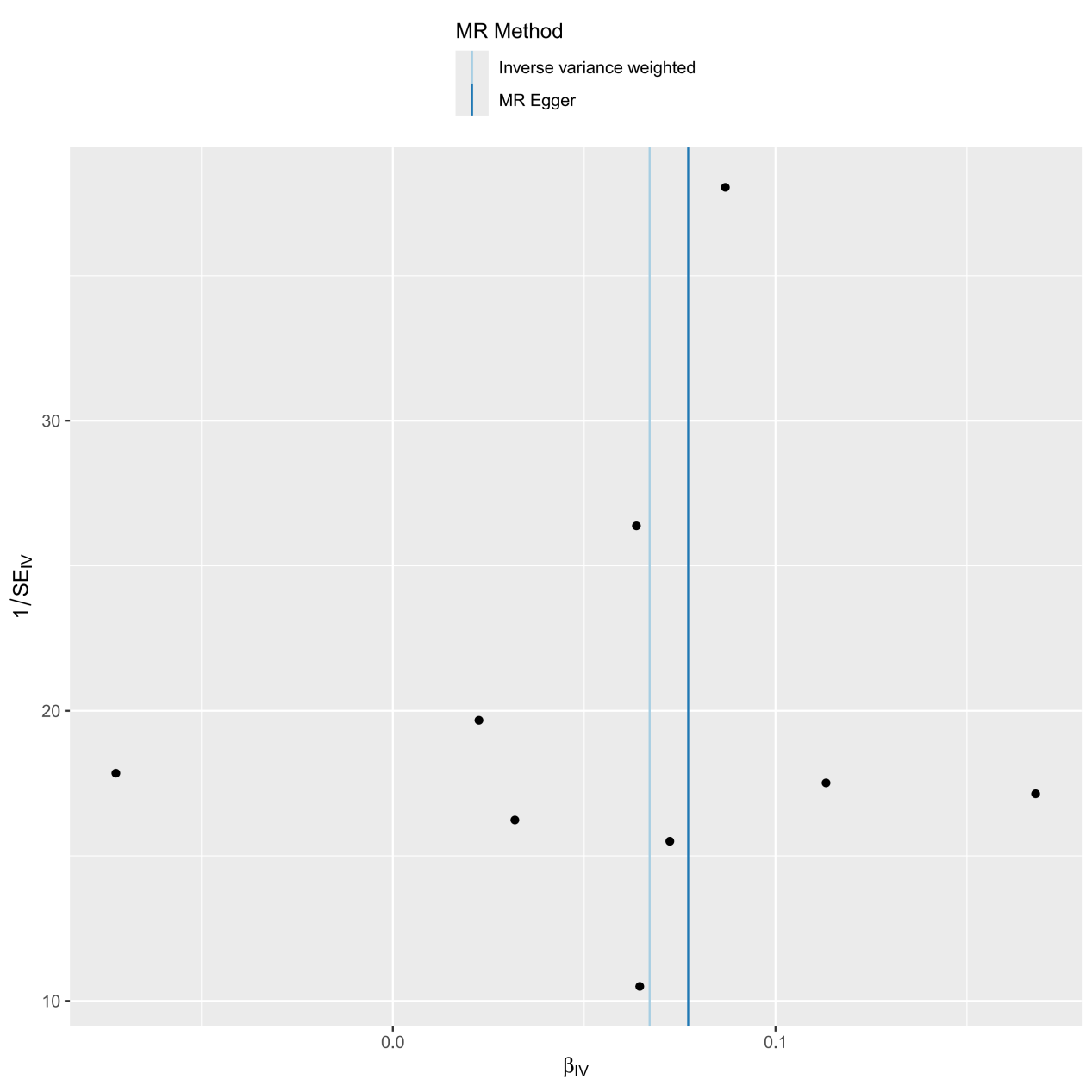


Gene 2: FABP2

Supplementary Figure 4. Leave-one-out analysis for the Mendelian randomization of FABP2.


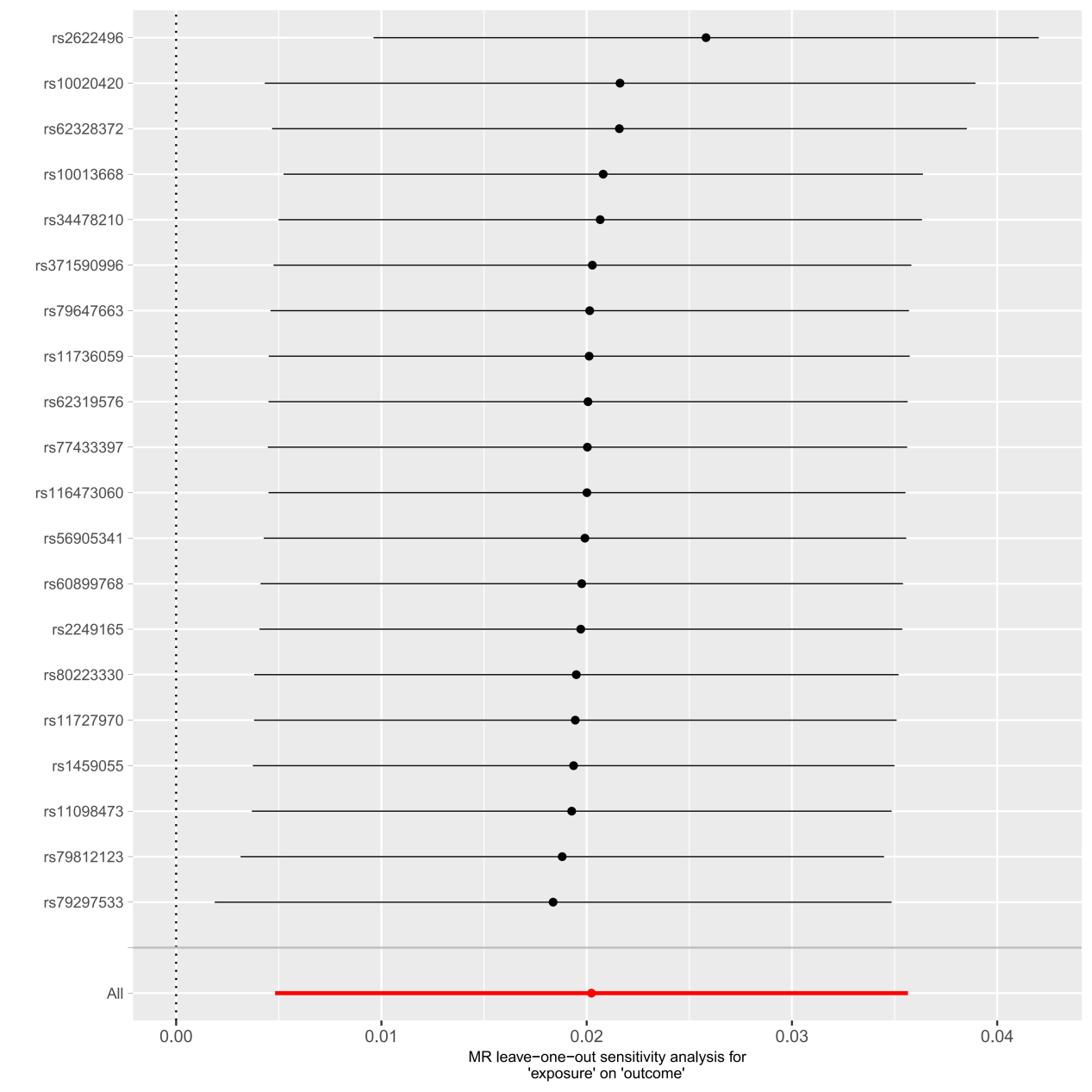


Supplementary Figure 5. Forest plot of the Mendelian randomization estimates for FABP2.


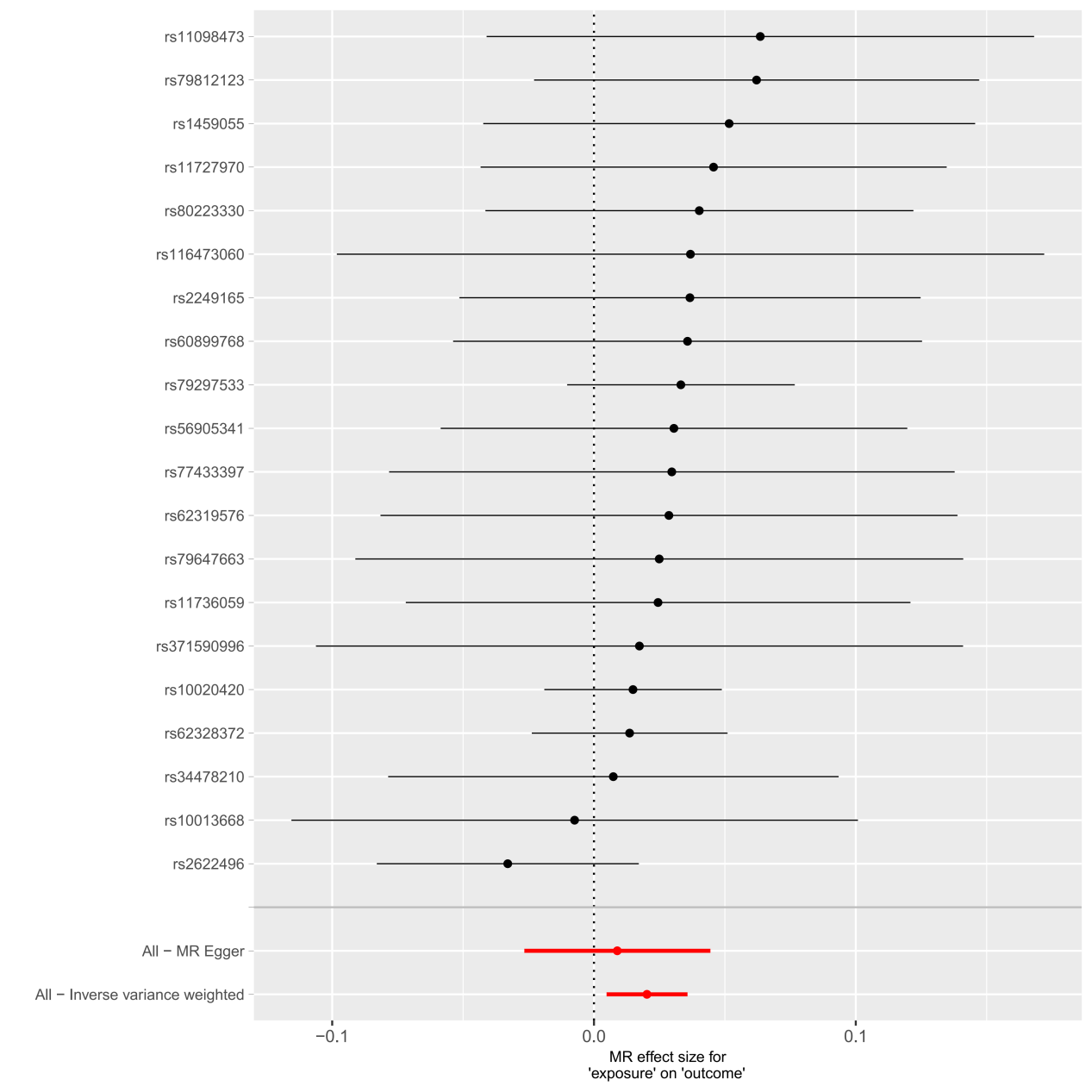


Supplementary Figure 6. Funnel plot for the Mendelian randomization of FABP2.


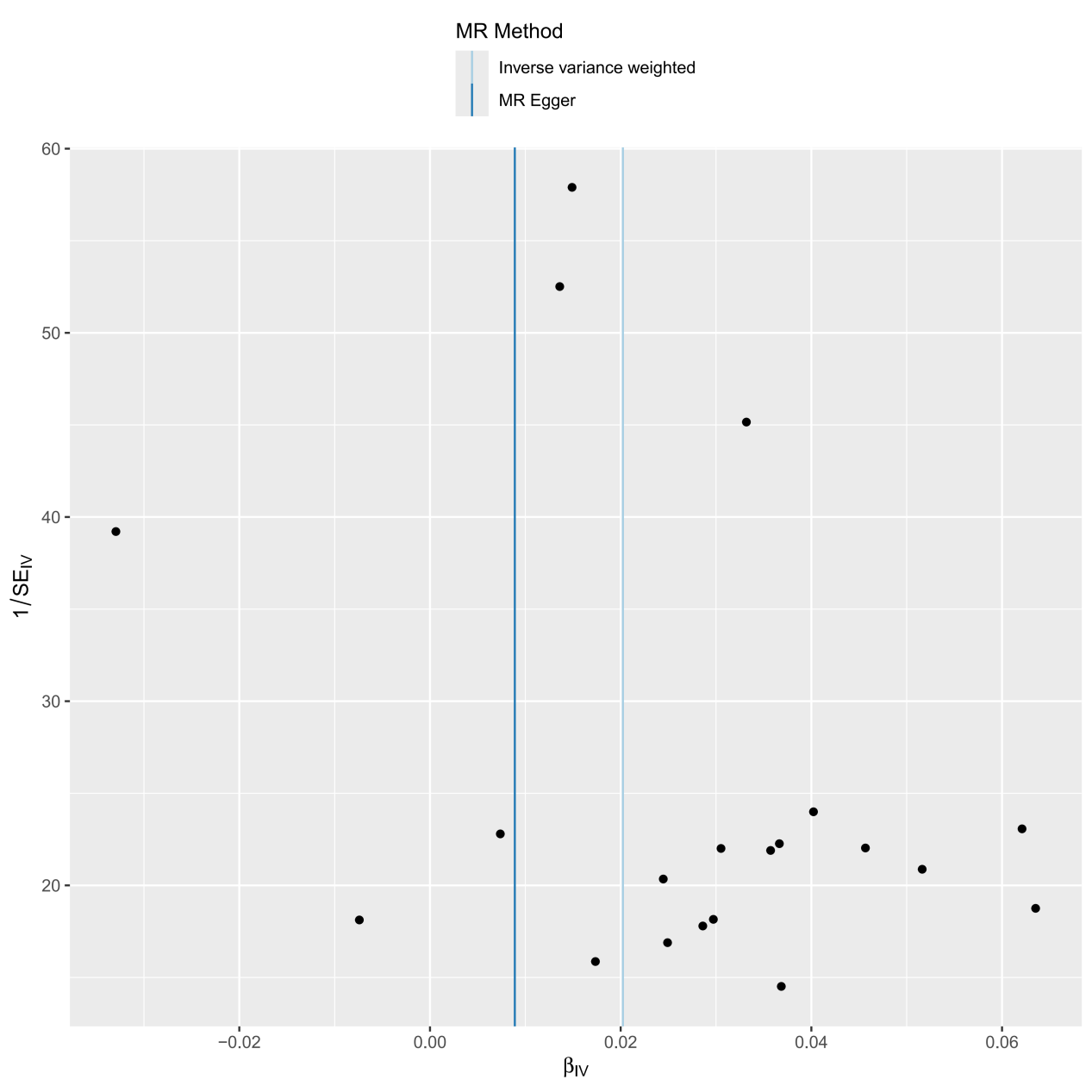


Gene 3: FDFT1

Supplementary Figure 7. Leave-one-out analysis for the Mendelian randomization of FDFT1.


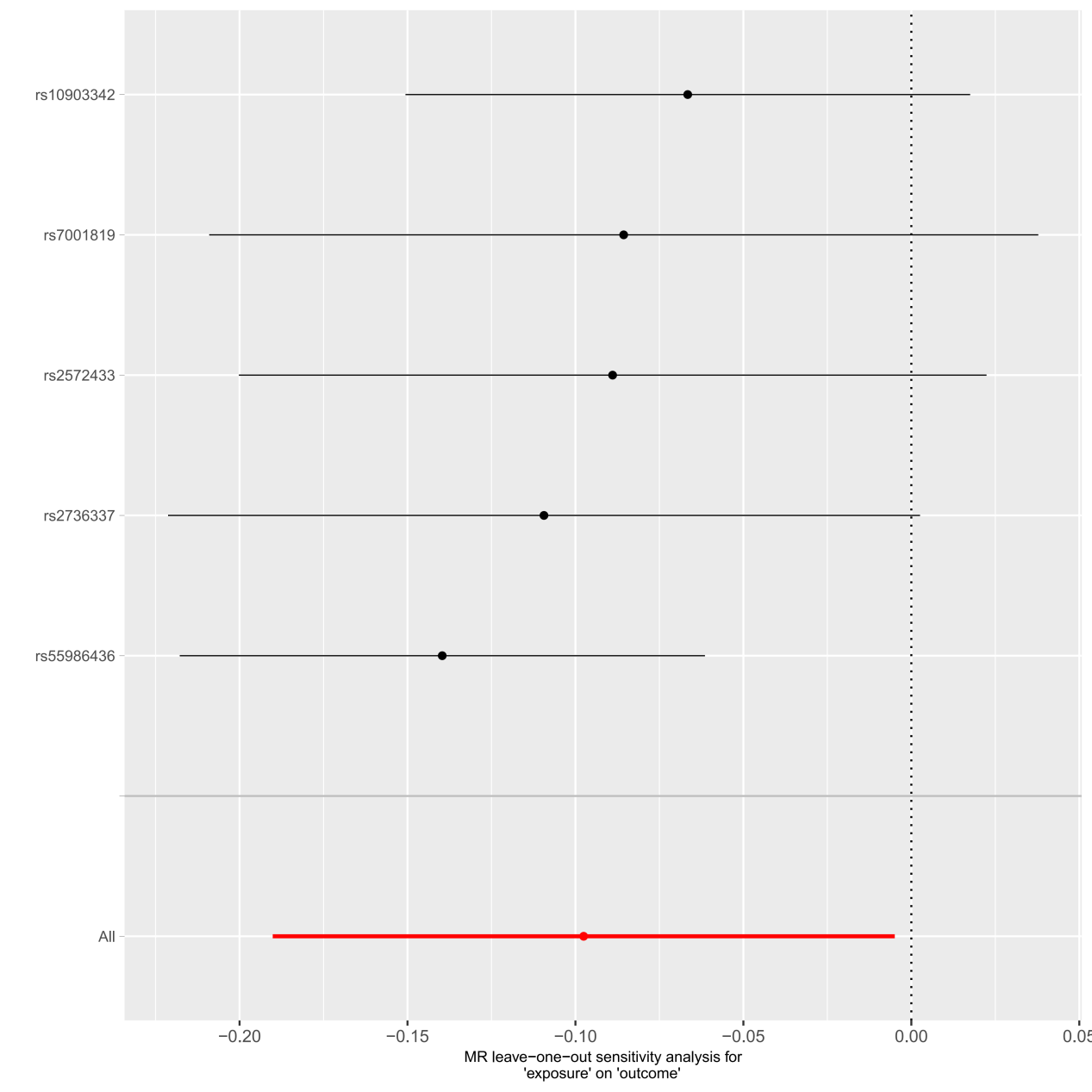


Supplementary Figure 8. Forest plot of the Mendelian randomization estimates for FDFT1.


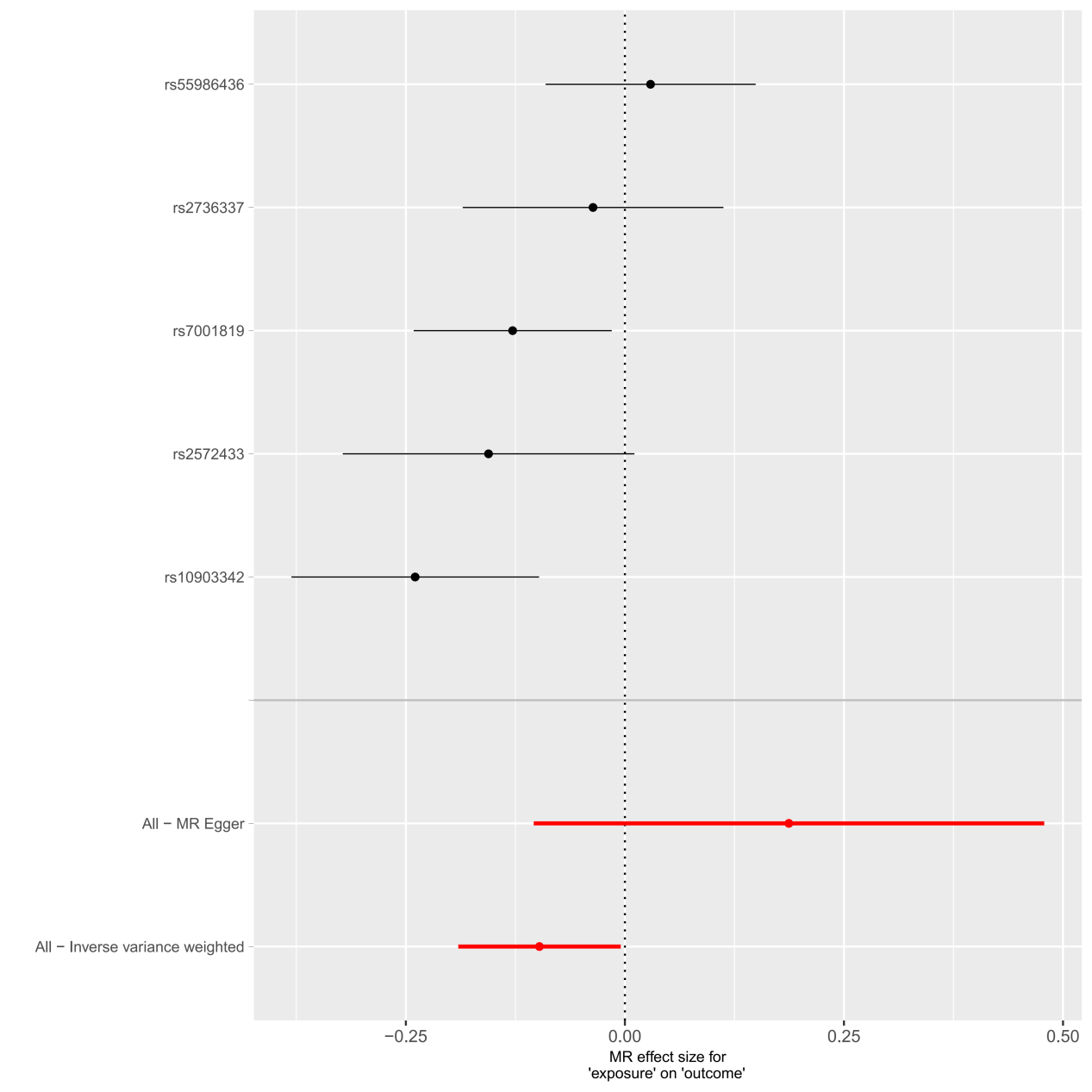


Supplementary Figure 9. Funnel plot for the Mendelian randomization of FDFT1.


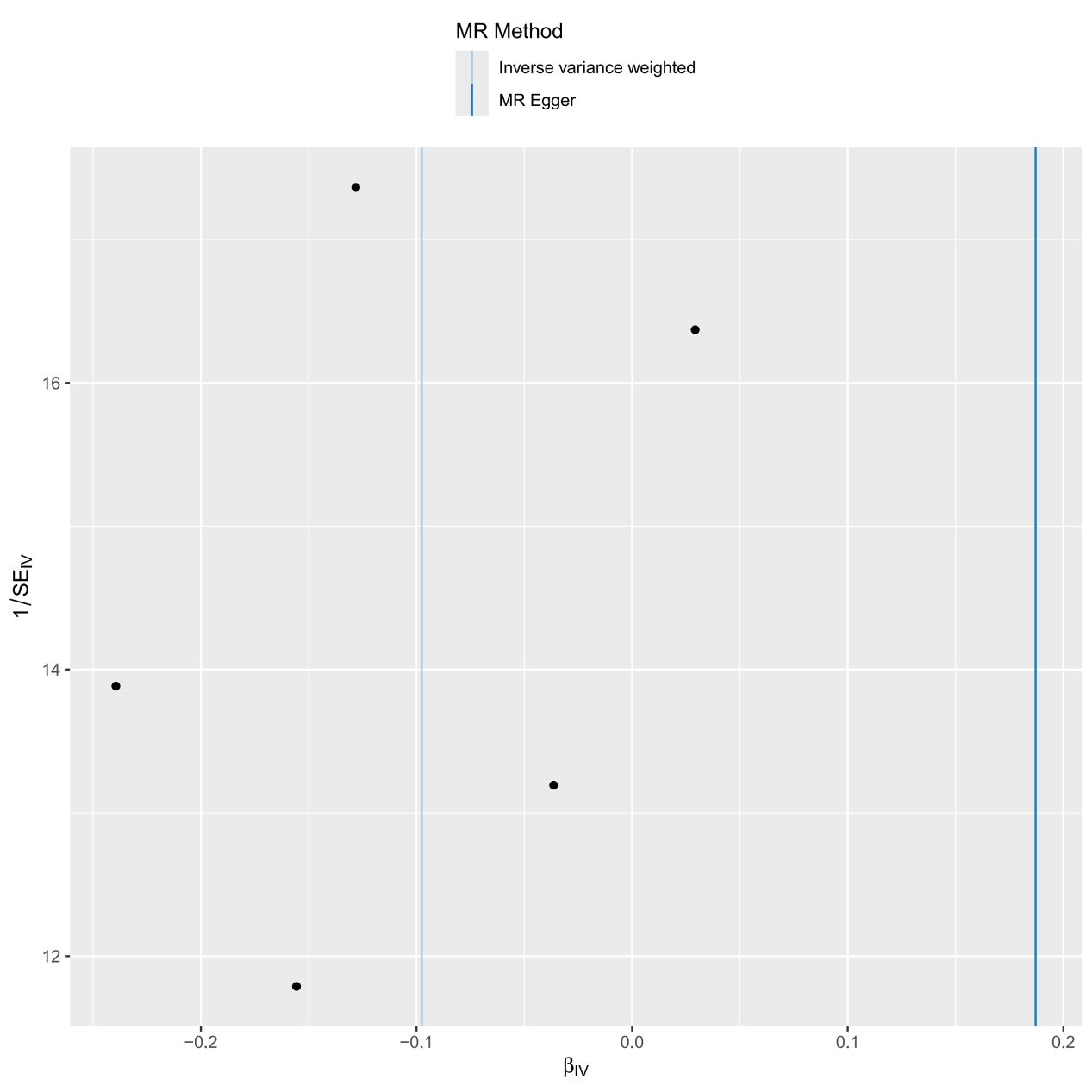


Gene 4: HINT1

Supplementary Figure 10. Leave-one-out analysis for the Mendelian randomization of HINT1.


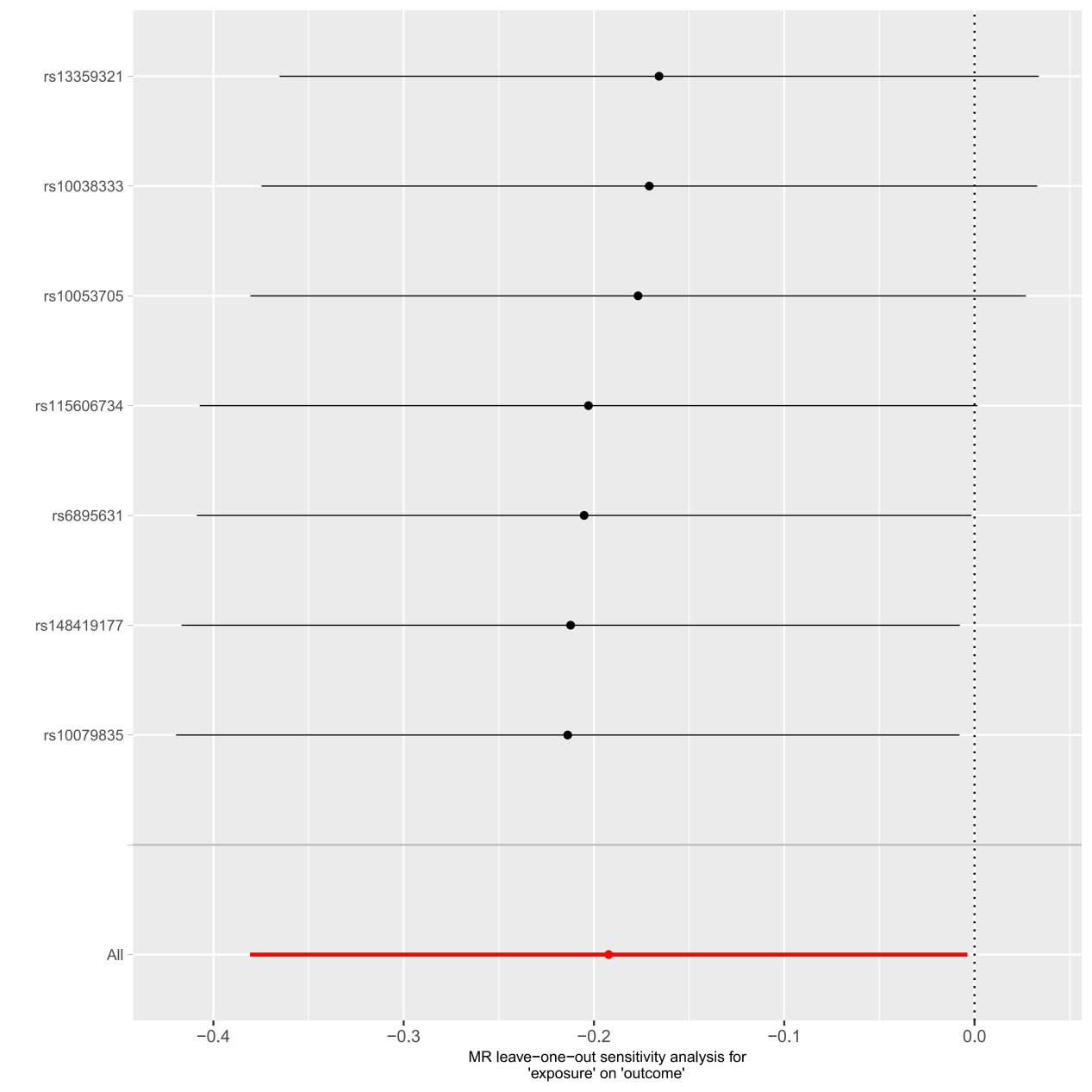


Supplementary Figure 11. Forest plot of the Mendelian randomization estimates for HINT1.


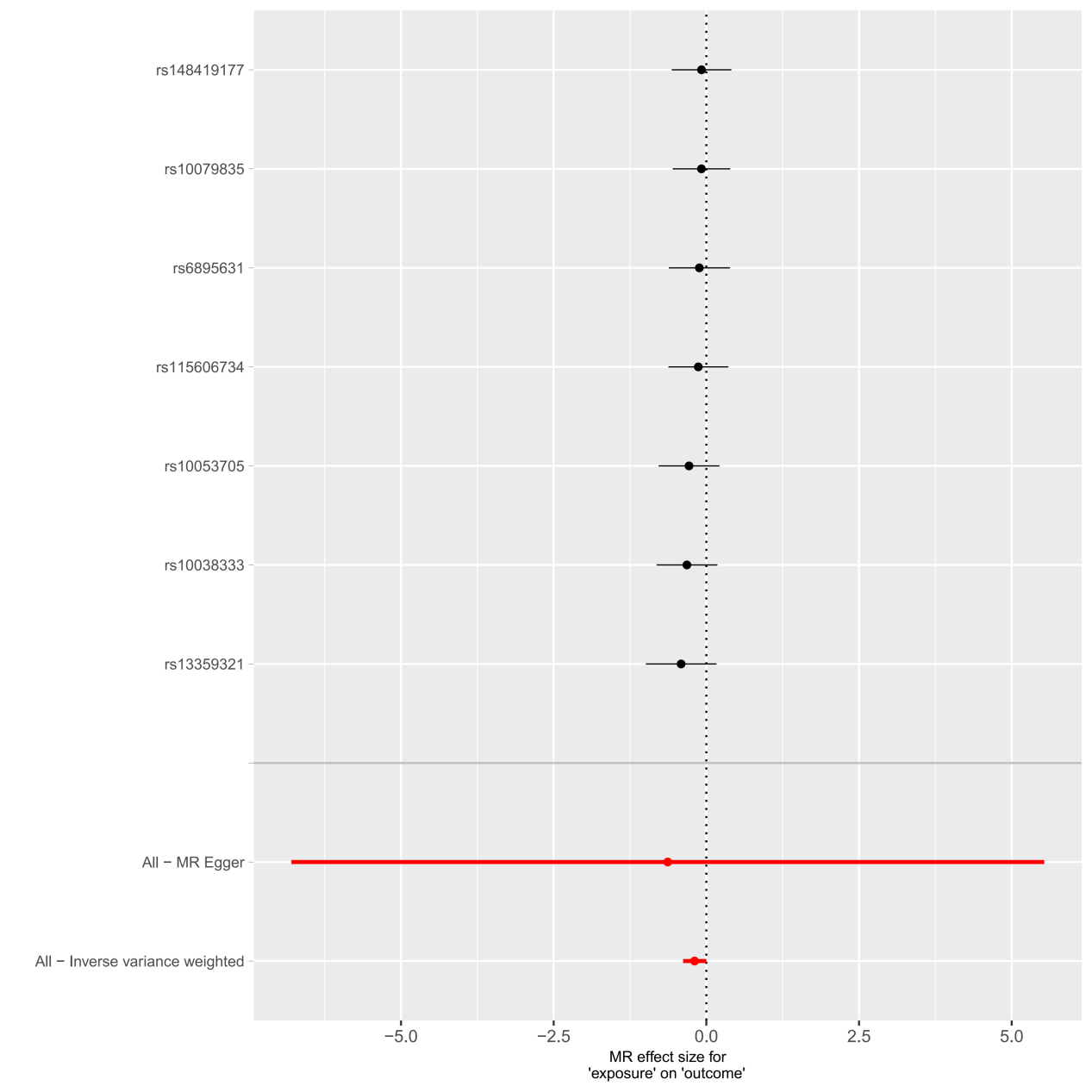


Supplementary Figure 12. Funnel plot for the Mendelian randomization of HINT1.


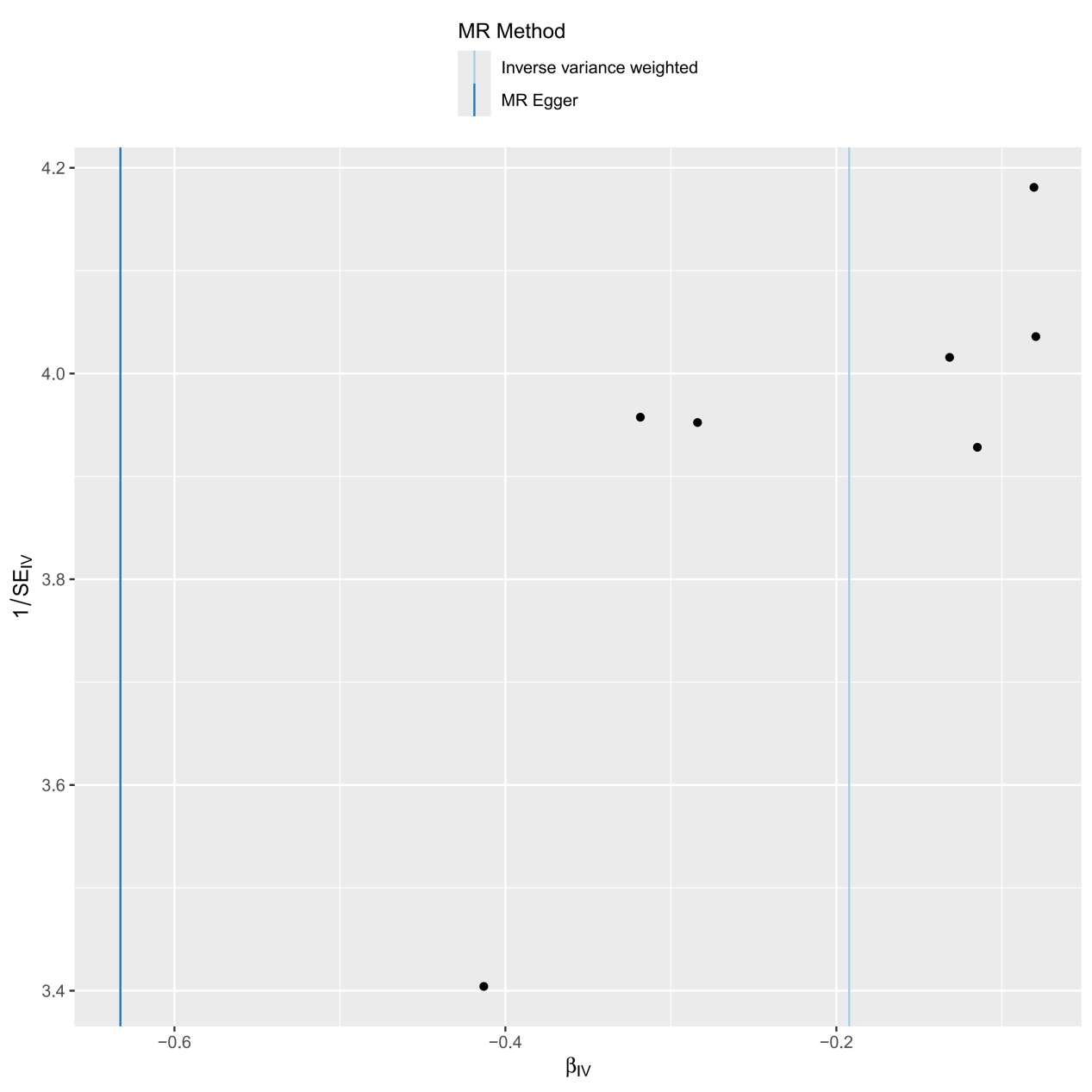


Gene 5: NDST1

Supplementary Figure 13. Leave-one-out analysis for the Mendelian randomization of NDST1.


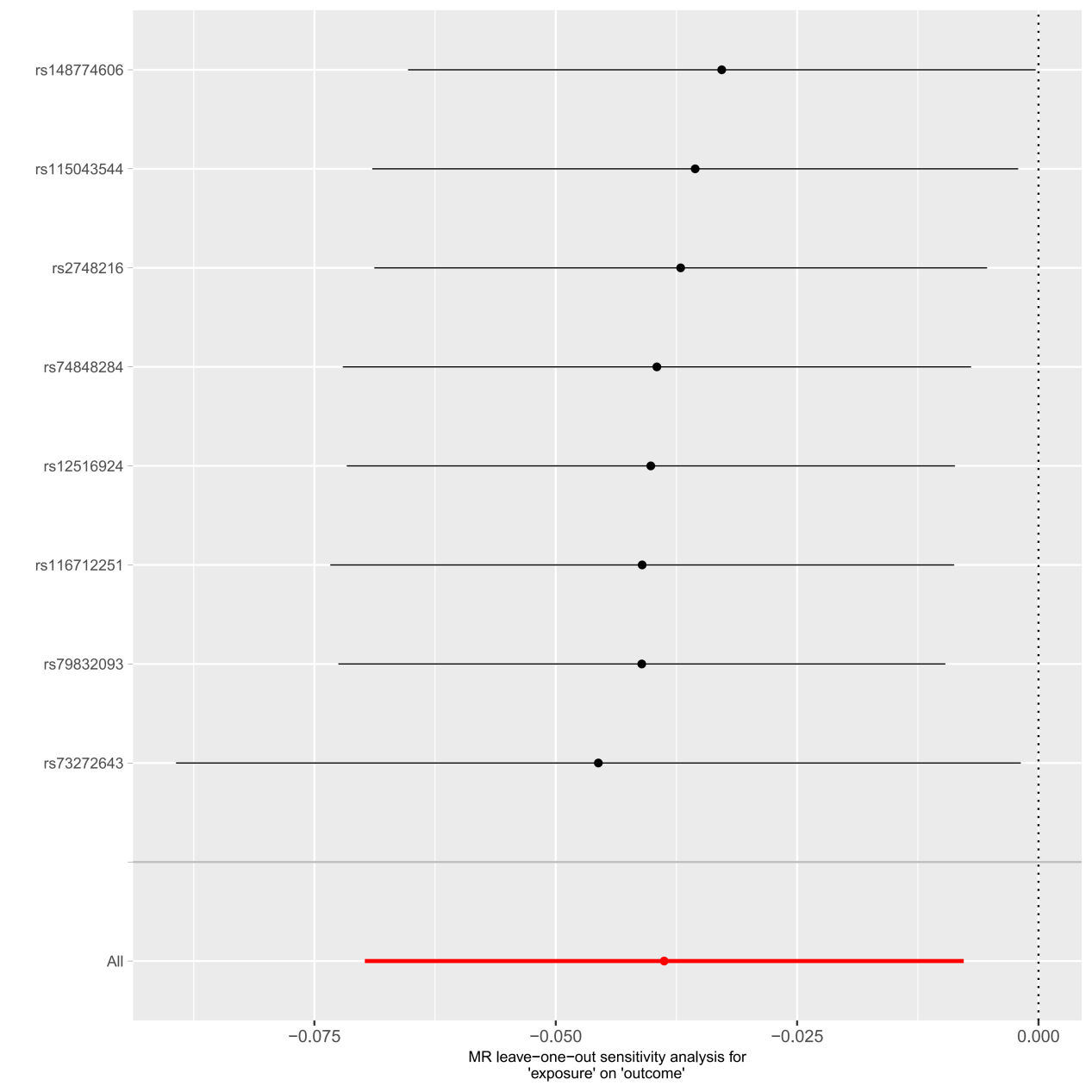


Supplementary Figure 14. Forest plot of the Mendelian randomization estimates for NDST1.


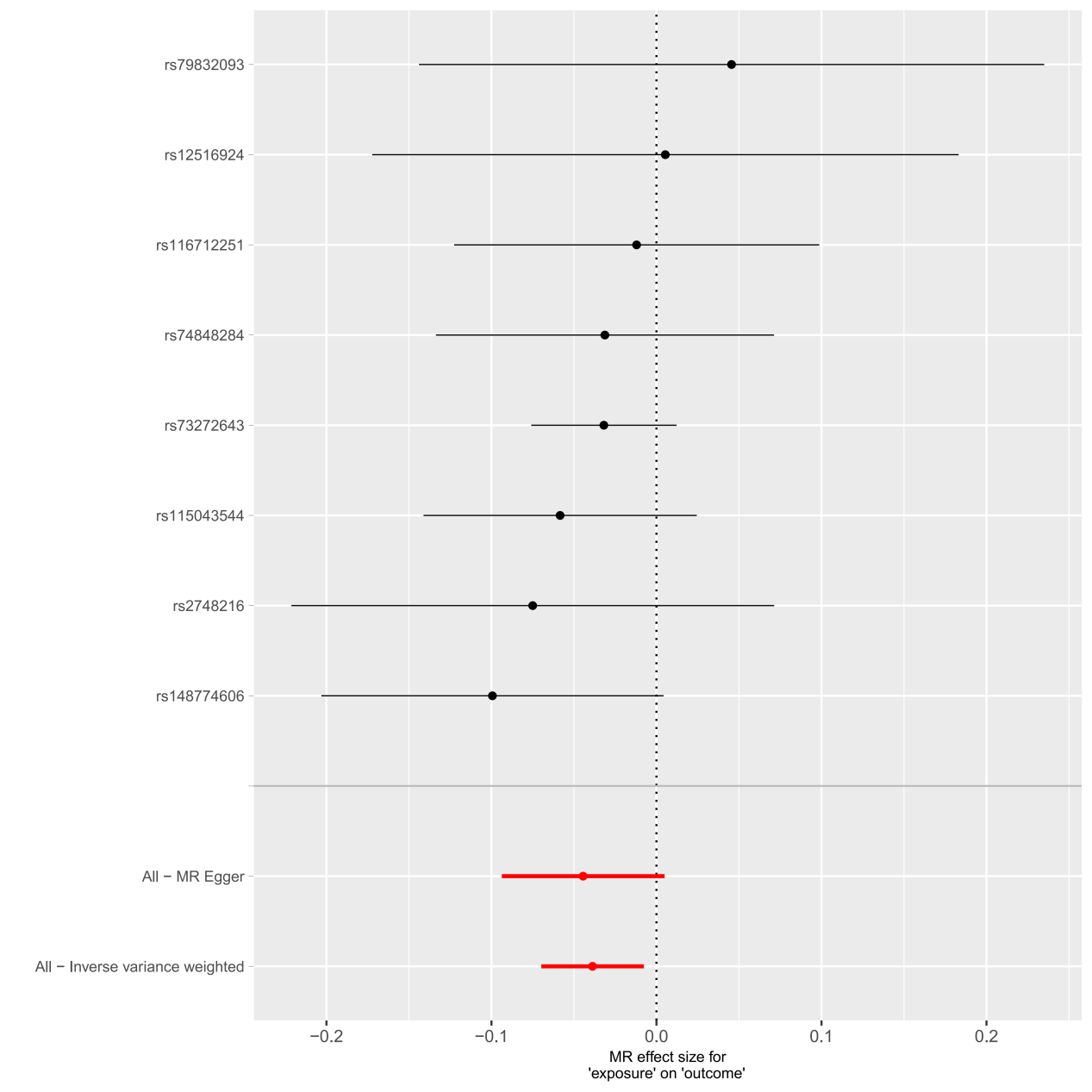


Supplementary Figure 15. Funnel plot for the Mendelian randomization of NDST1.


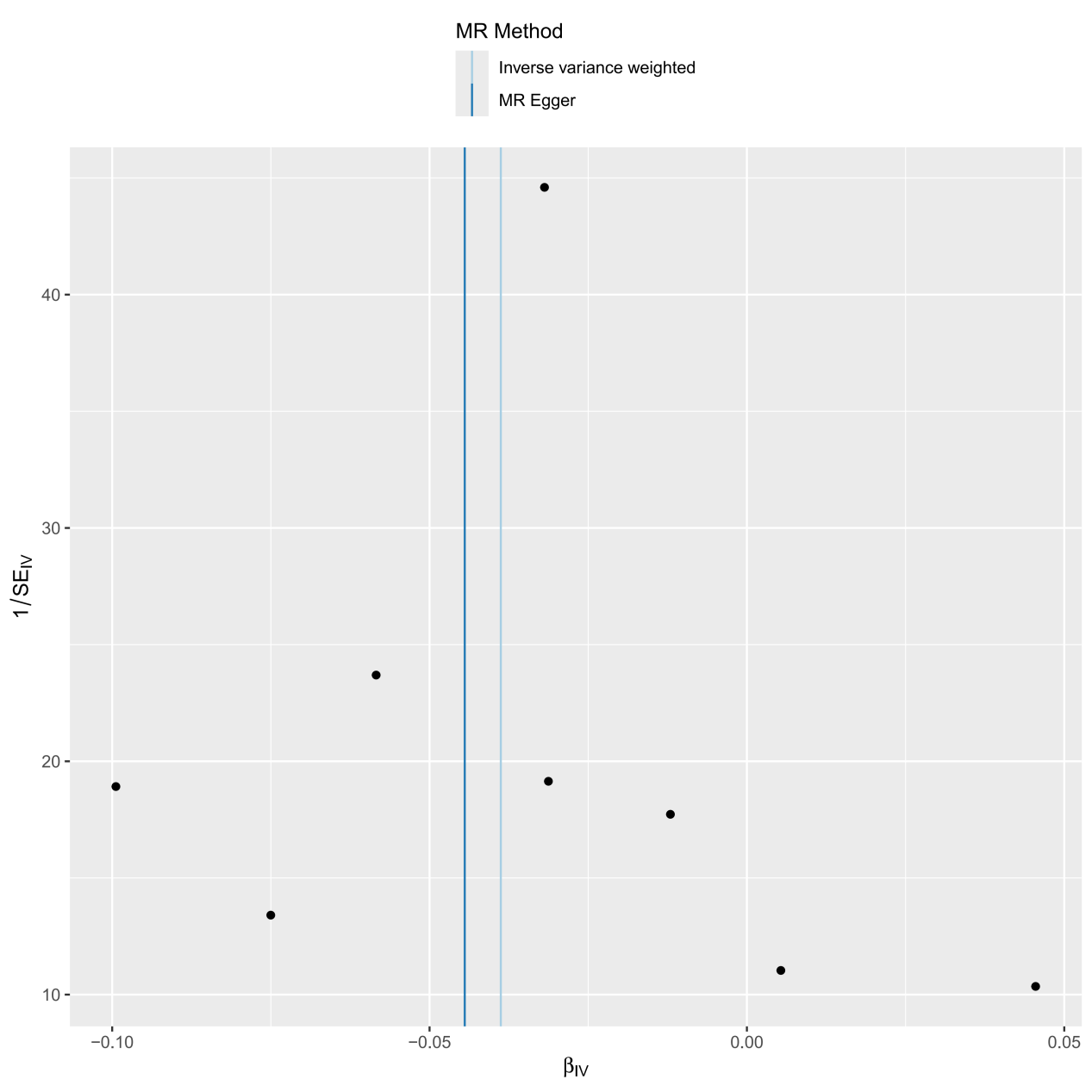


Gene 6: PTGER4

Supplementary Figure 16. Leave-one-out analysis for the Mendelian randomization of PTGER4.


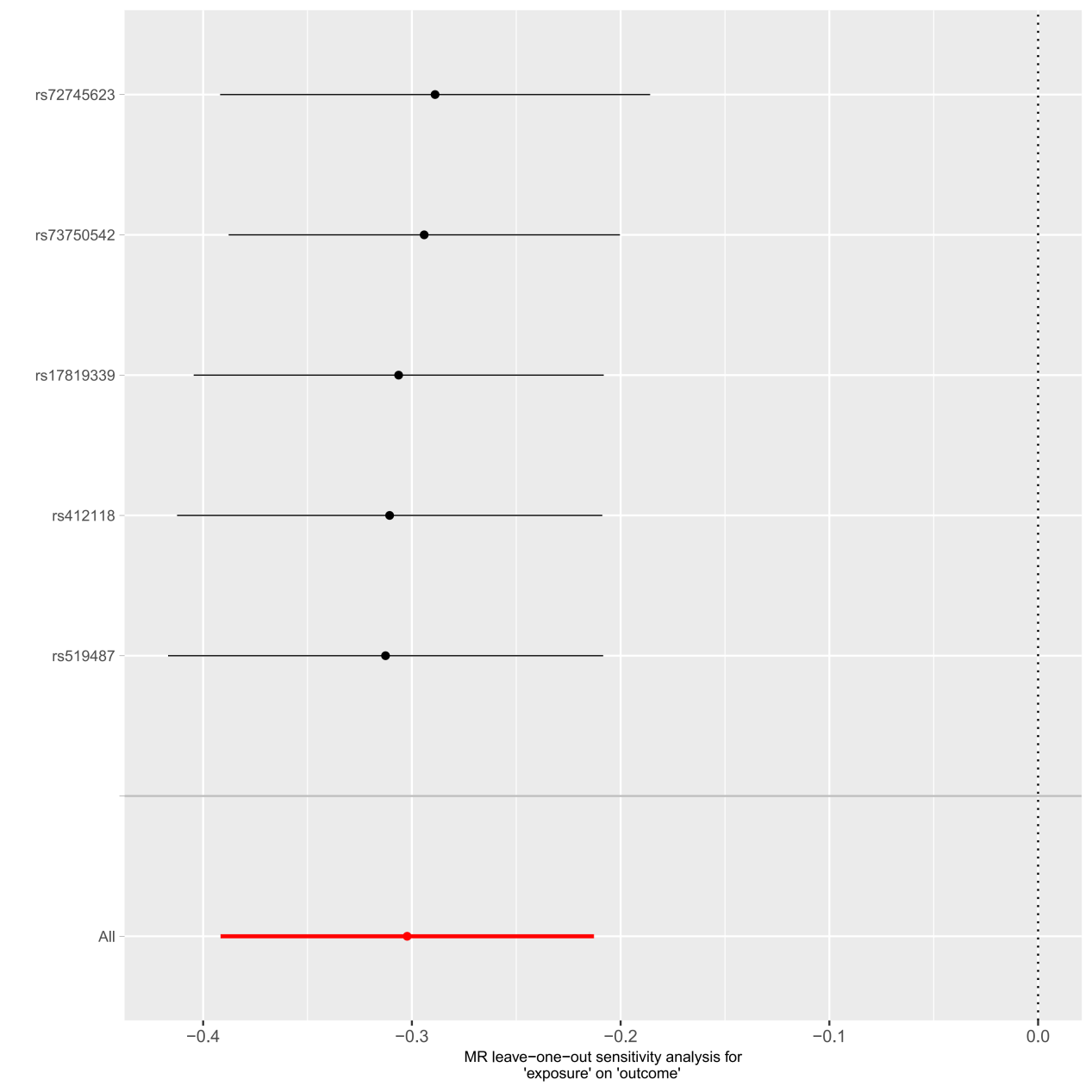


Supplementary Figure 17. Forest plot of the Mendelian randomization estimates for PTGER4.


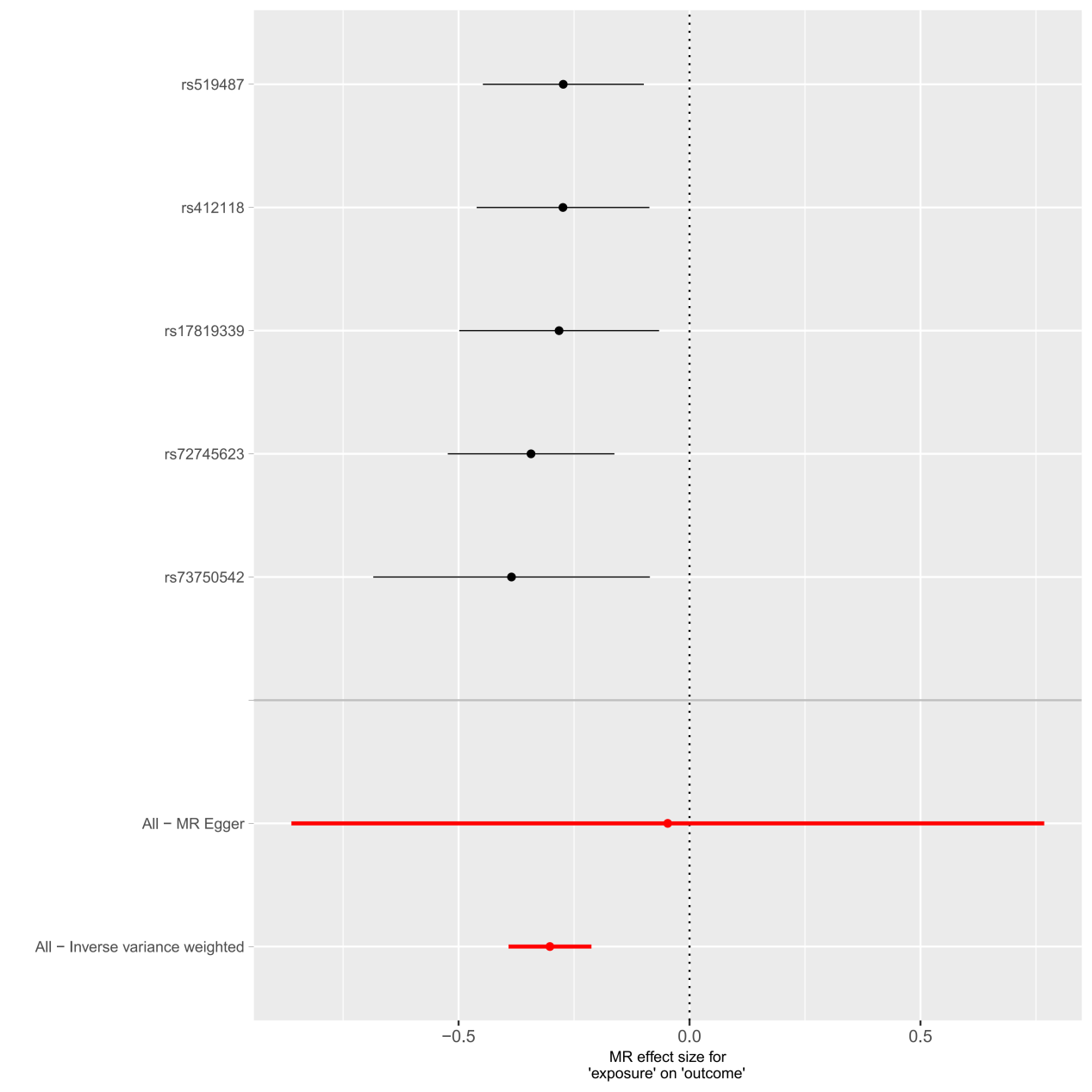


Supplementary Figure 18. Funnel plot for the Mendelian randomization of PTGER4.


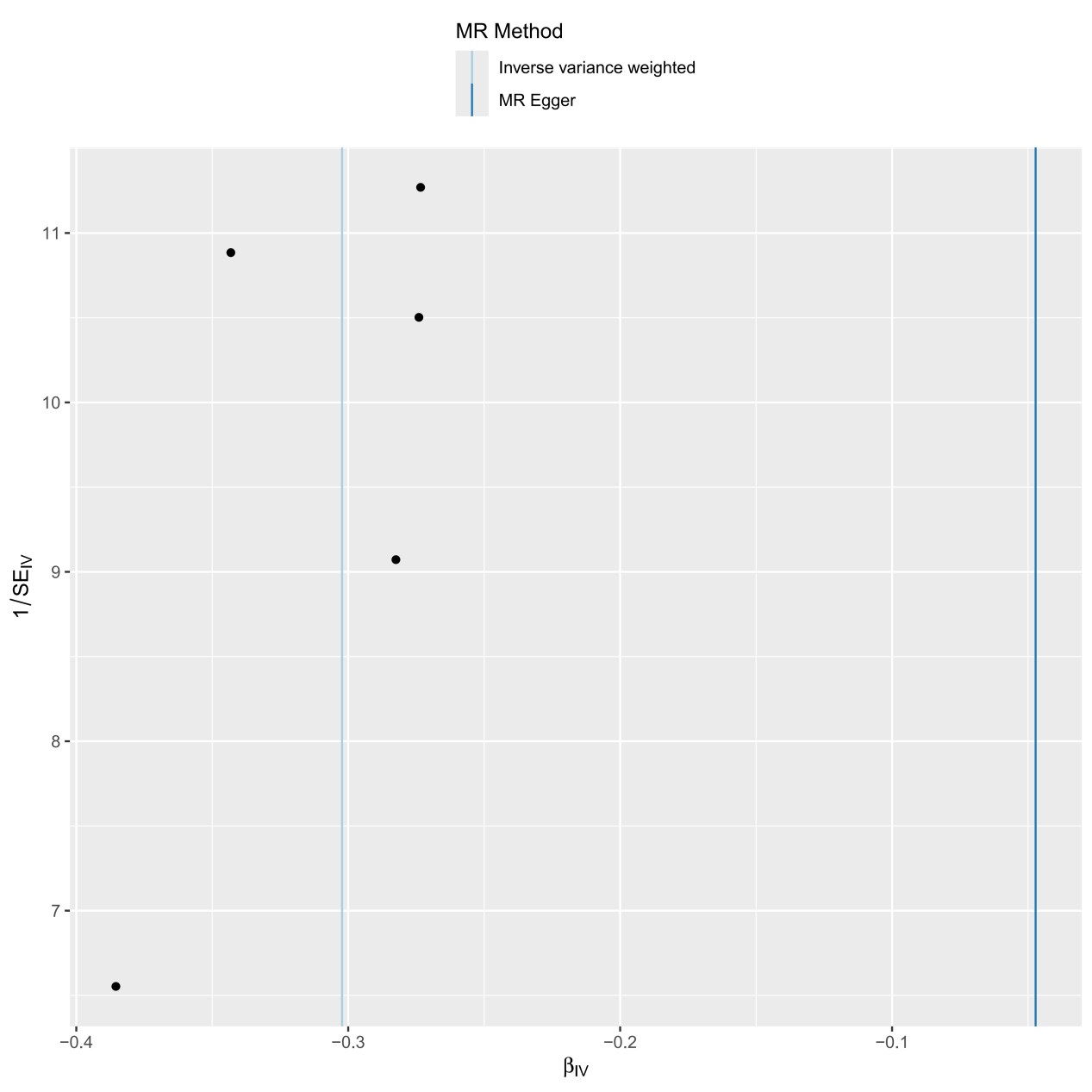

Supplement: Supplementary file 3 — Supplementary Material 3. [file 12958_2025_1508_MOESM3_ESM.docx]
